# Piezo1-mediated mechanotransduction regulates the translational activity, function and lung pathogenicity of group 2 innate lymphoid cells

**DOI:** 10.1038/s41392-025-02350-4

**Published:** 2025-08-21

**Authors:** MinYeong Lim, Seonjun Park, Yoon Ha Joo, Sung Eun Kim, Min Hee Ham, TaeSoo Kim, Kihyuck Kwak, Sung Joon Kim, Jung Chan Lee, Sung Ho Park, Hye Young Kim

**Affiliations:** 1 Laboratory of Mucosal Immunology, Department of Biomedical Sciences, Seoul National University College of Medicine, Seoul, 03080 South Korea; 2 Department of Life Science, Multitasking Macrophage Research Center, Ewha Womans University, Seoul, 03760 South Korea; 3 Department of Biological Sciences, Ulsan National Institute of Science and Technology (UNIST), Ulsan, 44919 South Korea; 4 Interdisciplinary Program in Bioengineering, Graduate School, Seoul National University, Seoul, 08826 South Korea; 5 Department of Neurology, Division of Sleep Medicine, Harvard Medical School and Beth Israel Deaconess Medical Center, Boston, MA 02215 USA; 6 Department of Physiology, College of Medicine, Seoul National University, Seoul, 03080 South Korea; 7 Department of Microbiology and Immunology, College of Medicine, Yonsei University, Seoul, 02447 South Korea; 8 Brain Korea 21 PLUS Project for Medical Science, College of Medicine, Yonsei University, Seoul, 03722 South Korea; 9 Institute for Immunology and Immunological Diseases, Yonsei University College of Medicine, Seoul, 03722 South Korea; 10 Wide River Institute of Immunology, College of Medicine, Seoul National University, Hongcheon, 25159 South Korea; 11 Department of Biomedical Engineering, College of Medicine, Seoul National University, Seoul, 08826 South Korea; 12 Institute of Medical and Biological Engineering, Seoul National University Medical Research Center, Seoul, 03080 South Korea; 13 Institute of Allergy and Clinical Immunology, Seoul National University Medical Research Center, Seoul, 03080 South Korea

**Keywords:** Innate immune cells, Inflammation, Translational immunology, Innate immunity

## Abstract

Group 2 innate lymphoid cells (ILC2s) are central effectors of type 2 immune responses in the lung; however, how mechanical cues regulate their function remains unclear. Here, we identified the mechanosensitive ion channel Piezo1 as a key regulator of ILC2 effector function through translational control. Piezo1 is highly expressed in murine and human ILC2s, and its activation by mechanical stress or the Piezo1 agonist, Yoda1 induces calcium influx, triggering mTOR signaling and selectively enhancing IL-13 protein production. Conditional deletion of Piezo1 in ILC2s reduced mTOR activation and puromycin incorporation, leading to impaired protein synthesis and attenuated lung inflammation and fibrosis in the IL-33, *Alternaria alternata*, and bleomycin models. scRNA-seq and scATAC-seq confirmed that Piezo1-deficient ILC2s retained *Il13* transcription and chromatin accessibility but presented translational suppression, as evidenced by protein‒mRNA interactions. Pharmacologic mTOR inhibition phenocopied Piezo1 loss, supporting the functional relevance of the Piezo1–mTOR axis. These findings demonstrate that Piezo1 functions as a mechanosensor that integrates biomechanical cues to regulate cytokine output via mTOR-mediated translation. Targeting Piezo1 signaling or its downstream effectors may provide therapeutic benefits in type 2 inflammation–associated lung diseases.

## Introduction

The lung is one of the most complex and dynamic organs in the human body and performs critical physiological functions such as gas exchange, mucociliary clearance, and immune surveillance.^1^ It is constantly exposed to airborne environmental challenges, including pollutants, allergens, and pathogens, while it processes thousands of liters of air each day.^2^ These respiratory dynamics subject the lung parenchyma—particularly the alveolar regions—to continuous mechanical forces such as shear stress, stretching, and pressure changes. These forces, although part of normal respiration, can pose a threat to the structural integrity of the lung and are known to modulate local immune responses.^3^ To counteract such stressors and maintain pulmonary homeostasis, the lung relies on a diverse network of structural and immune cells capable of detecting both external stimuli and internal biomechanical changes. Despite the increasing appreciation for the role of mechanical forces in lung physiology, the specific molecular sensors and signaling pathways that mediate immune cell responses to such forces remain incompletely understood.

Mechanotransduction—the biological process by which cells convert mechanical stimuli into biochemical signals—is essential for adapting cellular behaviors to dynamic physical environments.^3,4^ This process governs key aspects of development, tissue maintenance, and immunity across multiple organ systems. Within the immune system, both innate and adaptive cells respond to mechanical forces. For example, epithelial cell stretching promotes proliferation,^5^ whereas cyclic strain on dendritic cells (DCs) enhances their antigen-presenting capabilities.^6^ At the molecular level, mechanotransduction frequently involves the activation of mechanosensitive ion channels (MSICs), which detect membrane deformation and permit the influx of ions—primarily calcium ions (Ca²⁺)—into the cytoplasm.^7^ The resulting increase in intracellular Ca²⁺ can trigger cytoskeletal remodeling and initiate downstream pathways that regulate gene expression, protein translation, migration, and cytokine secretion. Among MSICs, Piezo1 has emerged as a central and evolutionarily conserved mechanosensor. It responds specifically to mechanical stimuli, such as stretch and shear stress, and has been implicated in numerous physiological processes, including vascular development, neural function, and immune regulation.^8–11^ In immune cells such as macrophages, DCs, and T cells, Piezo1 activation enhances motility, cytokine production, and T-cell priming, particularly within mechanically dynamic tissues such as the lung, heart, and tumor microenvironments.^10,12,13^ Piezo1 also enables T cells to sense fluid shear stress and enhances their activation during inflammation; notably, its deletion reduces disease severity in autoimmune models such as experimental autoimmune encephalomyelitis.^11^ These findings suggest that Piezo1 may serve as a critical link between mechanical cues and immune cell function, but whether Piezo1 exerts similar effects on innate lymphoid cells has not been fully explored.

In the lung, group 2 innate lymphoid cells (ILC2s) represent a key subset of immune effectors that respond rapidly to epithelial stress and damage. Unlike T and B lymphocytes, ILC2s lack antigen-specific receptors and instead rely on cytokine signals, particularly epithelial-derived alarmins such as IL-25, IL-33, and thymic stromal lymphopoietin (TSLP), to initiate activation.^14^ Once activated, ILC2s secrete hallmark type 2 cytokines—including IL-5, IL-9, and IL-13—which recruit eosinophils, activate macrophages, and enhance T-cell responses. These functions are essential for tissue repair following injury and for host defense against helminth infections. However, dysregulated or sustained ILC2 activation can drive pathological outcomes, such as airway hyperresponsiveness (AHR), excessive mucus production, and fibrotic remodeling, contributing to chronic lung diseases such as asthma and pulmonary fibrosis.^15,16^ Notably, ILC2s are tissue-resident and strategically positioned near epithelial surfaces where they may be directly exposed to mechanical forces associated with respiration. Despite their established role in lung inflammation, the extent to which mechanical cues modulate ILC2 activity, as well as the molecular pathways that govern such mechanosensitive responses, remains largely unknown.

To address this knowledge gap, we investigated whether ILC2s sense and respond to mechanical forces through Piezo1 and how this mechanotransduction influences their effector functions in inflammatory conditions. Using both murine and human systems, we demonstrated that ILC2s express functional Piezo1 channels. In vitro activation of Piezo1—either through applied mechanical stress or the Piezo1-specific agonist Yoda1—selectively enhanced IL-13 production without affecting IL-4 or IL-5. Mechanistically, Piezo1-mediated calcium influx activated the mammalian target of the rapamycin (mTOR) pathway, leading to increased *Il13* mRNA stability and translation. Deletion of Piezo1 in ILC2s impaired these translational programs, resulting in markedly reduced cytokine production. In disease models of lung inflammation and fibrosis, Piezo1 deficiency in ILC2s attenuated type 2 immune responses and improved pathological outcomes. These results identify Piezo1 as a mechanosensor that selectively programs ILC2 effector functions by promoting IL-13–biased translation through Ca²⁺–mTOR signaling. Our findings provide mechanistic insight into how ILC2s integrate physical and immunological cues within the lung and highlight Piezo1 as a potential therapeutic target in type 2 inflammatory lung diseases.

## Results

### Piezo1 expression and function in murine and human ILC2s

**Fig. 1:**
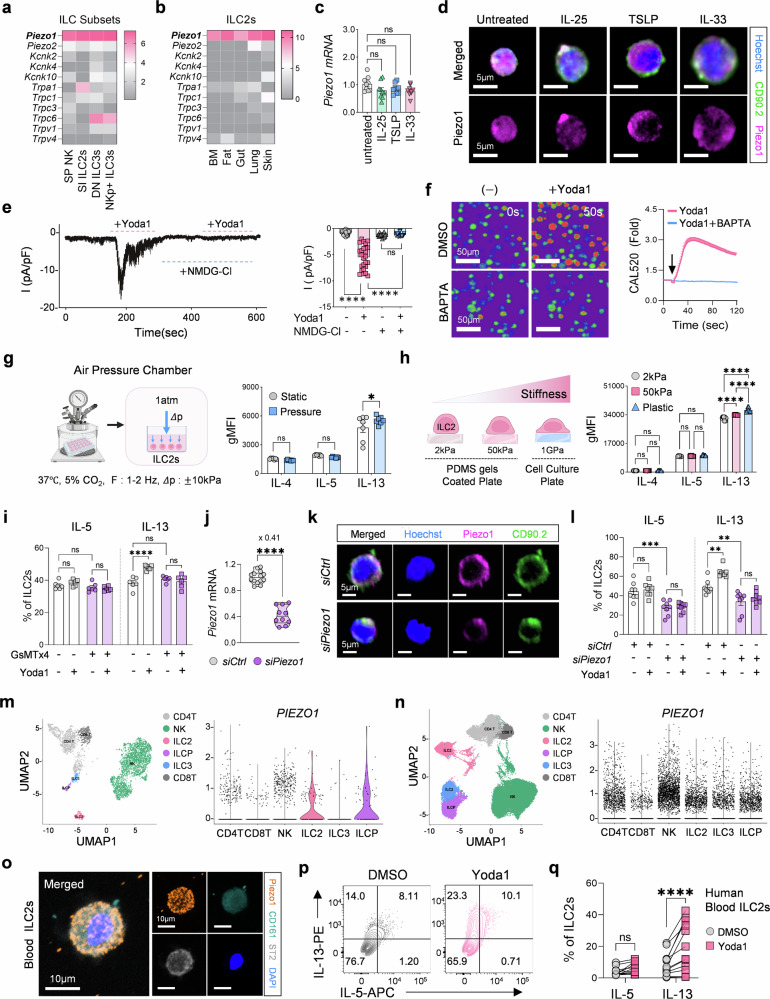
Expression and functional characterization of the mechanosensitive ion channel Piezo1 in ILC2s. **a** Gene expression of mechanosensitive ion channels across ILC subsets from the ImmGen database. **b** Expression of *Piezo1* in tissue-resident ILC2s from published bulk RNA-seq datasets (Roberto et al.). **c** *Piezo1* mRNA expression in ILC2s stimulated with IL-25, IL-33, or TSLP (*n* = 9). **d** Representative immunofluorescence images showing Piezo1 localization in alarmin-stimulated ILC2s (Piezo1: magenta, CD90.2: green, and Hoechst; scale bars = 5 µm). **e** Representative traces and quantification of Yoda1-induced membrane currents in ILC2s at –80 mV; currents were abolished in NMDG-Cl bath solution (*n* = 26). **f** Live-cell calcium imaging before and after Yoda1 stimulation (±BAPTA) via CAL-520 AM (*n* = 68–98) (scale bars = 50 µm). **g** Schematic of the cyclic air pressure (CAP) chamber and flow cytometry analysis of type 2 cytokine production in ILC2s under static vs. CAP conditions (*n* = 7). **h** Schematic of ILC2 culture on PDMS hydrogels (2 kPa and 50 kPa) or plastic plates and flow cytometry analysis of type 2 cytokine production in PDMS- or plastic-cultured ILC2s (*n* = 8). **i** Intracellular IL-5 and IL-13 levels in ILC2s stimulated with Yoda1 and/or the Piezo1 inhibitor GsMTx4 (*n* = 7). **j** Validation of *Piezo1* mRNA knockdown by siRNA in ILC2s (*n* = 10-14). **k** Representative immunofluorescence images of Piezo1 expression following siRNA transfection. (Piezo1: magenta, CD90.2: green, and Hoechst, Scale bars = 5 µm). **l** Intracellular IL-5 and IL-13 levels in *siRNA*-treated ILC2s stimulated with ± Yoda1 (*n* = 8). *PIEZO1* expression across immune cell subsets from public human fetal lung scRNA-seq datasets by He. (**m**) and Barnes et al. (**n**). **o** Representative images of Piezo1 immunofluorescence in human peripheral blood–derived ILC2s. (Piezo1: orange, CD161: green, ST2: white, and DAPI, Scale bars = 10 µm). **p** Representative flow cytometry plots showing IL-5⁺ and IL-13⁺ human ILC2s with or without Yoda1 stimulation. **q** Quantification of IL-5⁺ and IL-13⁺ in human ILC2s (*n* = 12). Statistical significance was determined via the Mann–Whitney U test, one-way ANOVA, two-way ANOVA, or paired t test, as appropriate. The data are representative of or pooled from at least two or three independent experiments and are presented as the means ± SEMs. **P < 0.01, ***P < 0.001, ****P < 0.0001; ns, not significant

We initially assessed Piezo1 expression across innate lymphoid cell (ILC) subsets, including ILC2s, NK cells, and ILC3s, via bulk RNA-seq data from the Immgen database (Fig. 1a). Our analysis revealed that *Piezo1* is broadly expressed across all ILC subsets. In addition, other public bulk RNA-seq data from Roberto et al.^17^ confirmed that ILC2s exhibit consistent expression across various tissues, such as bone marrow, fat, gut, lung, and skin (Fig. 1b). In contrast, other mechanosensitive ion channels (MSICs) were minimally expressed, suggesting that Piezo1 predominantly mediates MSIC activity in ILC2s (Supplementary Fig. 1a–c). Additionally, functional assays indicated that other potassium channels had a minimal effect on ILC2s (Supplementary Fig. 1d–f).

Next, we investigated whether Piezo1 expression is regulated upon stimulation with alarmins (IL-25, -33, and TSLP). Despite robust activation by these alarmins, *Piezo1* mRNA levels remained unchanged (Fig. 1c), and immunofluorescence revealed prominent localization of Piezo1 on the plasma membrane of ILC2s (Fig. 1d). Whole-cell patch-clamp assays confirmed that Yoda1, a Piezo1-specific agonist, induced inward currents at −80 mV,^18^ which were abolished under cation removal conditions (Fig. 1e). Consistently, calcium-sensitive CAL-520 AM-based intracellular calcium analysis demonstrated that Yoda1 stimulation induced significant Ca²⁺ influx, which was completely blocked by the calcium chelator BAPTA (Fig. 1f, Supplementary Fig. 1g, h, and Supplementary Movies 1–6). To explore Piezo1 function under mechanical stimulation, we employed two distinct models: a custom-made cyclic air pressure (CAP) chamber that mimics respiratory pressures and a defined-stiffness (2 and 50 kPa) PDMS hydrogel^19^ (Fig. 1g, h and Supplementary Fig. 2a-f). In both models, mechanical stimulation selectively enhanced IL-13 production without affecting IL-4 or IL-5 expression or compromising cell viability (Fig. 1g, h and Supplementary Fig. 2d–f). These results indicate that mechanical forces can regulate cytokine production in ILC2s.

To directly evaluate the role of Piezo1, we treated ILC2s with Yoda1. Yoda1 stimulation increased cell proliferation and increased cell size without inducing apoptosis (Supplementary Fig. 3a-d), and it selectively increased IL-13 levels but not IL-4 or IL-5 levels, particularly in IL-33-primed ILC2s (Supplementary Fig. 3e). Kinetics analysis confirmed that the IL-13 concentration progressively increased over time, whereas the IL-5 concentration remained unchanged (Supplementary Fig. 3f, g). Furthermore, Yoda1-induced IL-13 production was observed in adipose tissue-derived ILC2s (Supplementary Fig. 3h), suggesting a conserved regulatory role in ILC2s across different tissues. To validate Piezo1-mediated IL-13 production in ILC2s, we applied GsMTx4, a Piezo1 inhibitor.^20^ GsMTx4 blocked Yoda1-induced Ca²⁺ influx (Supplementary Fig. 1i) and IL-13 upregulation in ILC2s without affecting IL-5 production (Fig. 1i). Similarly, *Piezo1* knockdown with *siRNA* abrogated Yoda1-induced Ca²⁺ influx (Supplementary Fig. 1j) and IL-13 production in ILC2s, whereas *Il5* and *Il13* mRNA levels remained unchanged (Fig. 1j–l and Supplementary Fig. 3i, j).

Finally, we examined *PIEZO1* expression and function in human lung ILC2s via public single-cell RNA-seq datasets.^21,22^ Human ILC2s, ILC3s, and ILCPs presented detectable *PIEZO1* expression (Fig. 1m, n), whereas *PIEZO2* and other MSICs presented minimal expression (Supplementary Fig. 4a–d). Immunofluorescence analysis confirmed membrane-localized Piezo1 expression in peripheral blood-derived human ILC2s (Fig. 1o and Supplementary Fig. 4e). Consistent with our murine data, Yoda1 stimulation selectively increased IL-13 production in human ILC2s (Fig. 1p, q), with minimal changes in viability and in CD161, CD127, and ST2 expression (Supplementary Fig. 4f, g). Collectively, these results indicate that Piezo1 functions as a key mechanosensitive ion channel in murine and human ILC2s, integrating mechanical and chemical signals to selectively promote IL-13 production, thereby potentially regulating the type 2 immune response in the lung.

### Piezo1 activation promotes IL-13 translation in ILC2s

**Fig. 2:**
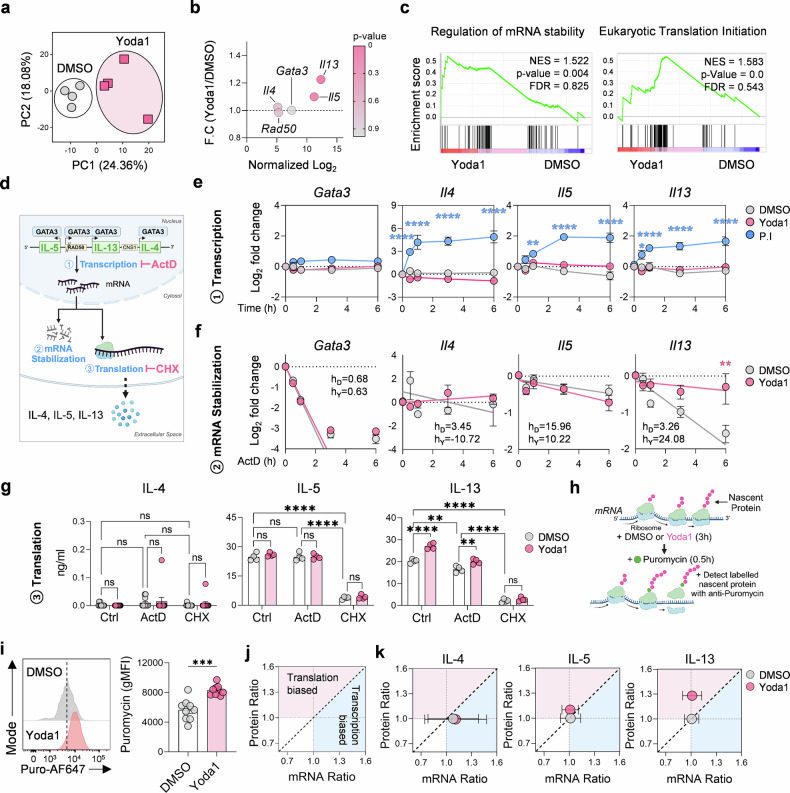
Piezo1 promotes IL-13 translation in ILC2s. **a** Principal component analysis (PCA) of bulk RNA-seq data comparing the transcriptomes of Yoda1- and DMSO-treated ILC2s (*n* = 4). **b** Differential expression of type 2 cytokine-associated genes in Yoda1-treated ILC2s. **c** Gene set enrichment analysis (GSEA) identifying pathways related to translation and *mRNA* stability enriched in Yoda1-treated ILC2s. **d** Schematic of type 2 cytokine gene loci and their transcriptional regulation by Gata3 and role of actinomycin D (ActD) and cycloheximide (CHX). **e** qPCR analysis of *Il4, Il5, Il13, Gata3*, and *Rad50* in ILC2s treated with DMSO (control), Yoda1 or PMA+ionomycin (P.I.) (*n* = 6–15). **f** *mRNA* decay curves following ActD treatment with or without Yoda1, showing increased *Il13* mRNA stability (*n* = 11); half-lives were calculated via a one-phase decay model. **g** Representative IL-13 and IL-5 protein levels in ILC2s treated with DMSO or Yoda1 ± ActD or CHX determined via ELISA (*n* = 4). **h** Schematic of the puromycin incorporation assay used to measure active translation. **i** Representative plots and quantification of puromycin^+^ ILC2s ± Yoda1 measured via flow cytometry (*n* = 9). **j** Schematic of cytokine transcription and translation phenotyping, with *mRNA* (x-axis, qPCR) and protein (y-axis, ELISA) ratios used to compare Yoda1 to DMSO. **k** Representative transcription and translation profiles of IL-4, IL-5, and IL-13 in ILC2s stimulated with DMSO or Yoda1 (*n* = 3). Statistical significance was determined via the Mann–Whitney U test, two-way ANOVA, or one-phase decay test for calculating the mRNA half-life, as appropriate. The data are representative of or pooled from at least two or three independent experiments and are presented as the mean ± SEM. **P < 0.01, ***P < 0.001, ****P < 0.0001

Typically, ILC2 activation induces both IL-5 and IL-13.^23^ However, our findings suggest that Piezo1 activation preferentially enhances IL-13 synthesis, indicating a bias toward IL-13 at the level of protein translation rather than transcription. To validate this, Yoda1-treated murine lung ILC2s were subjected to bulk mRNA-seq, which revealed unique transcriptional profiles compared with those of vehicle (DMSO-treated) controls (Fig. 2a) and differential expression of select genes (Supplementary Fig. 5a). Among the type 2 cytokine locus-associated genes, both *Il13* and *Il5* presented slight increases in mRNA expression (fold change | F. C | > 1.2 and |F. C | > 1.1, respectively), whereas *Il4*, *Rad50*, and *Gata3* expression remained unchanged (Fig. 2b). Further analysis confirmed that Yoda1 did not broadly affect ILC2 signature gene expression (Supplementary Fig. 5b), except for minor reductions in CD25 and CD127 expression and a subtle upregulation of PD-1 expression (Supplementary Fig. 5c–e). Accordingly, gene set enrichment analysis (GSEA) highlighted pathways related to mRNA stability and translation initiation, suggesting that Piezo1 signaling impacts posttranscriptional regulation (Fig. 2c).

To further dissect this translational reprogramming, we explored its effects across three gene expression stages: transcription, mRNA stability, and translation. The type 2 cytokine genes (*Il4*, *Il5*, and *Il13*) are clustered on chromosome 5 in humans and chromosome 11 in mice, and all of these genes are regulated by the transcription factor Gata3 (Fig. 2d).^24,25^ Initial qPCR analyses revealed no substantial transcriptional changes in *Gata3*, *Il4*, *Il5*, *Il13*, or *Rad50* upon Yoda1 stimulation, whereas PMA+ionomycin (P.I.) robustly induced type 2 cytokine *mRNA* expression but reduced *Rad50* (Fig. 2e and Supplementary Fig. 6a). Investigating mRNA stability via actinomycin D (ActD) treatment revealed that Yoda1 significantly stabilized *Il13* mRNA (h_D_ = ~3.26 h → h_Y_ = ~24.08 h), whereas other cytokine transcripts remained unaffected (Fig. 2f and Supplementary Fig. 6b). To test whether Piezo1 also regulates IL-13 translation, we examined protein production under transcriptional (ActD) and translational (cycloheximide (CHX)) blockade. While ActD reduced IL-13 but not IL-5 levels, Yoda1 could still partially increase IL-13 production under transcriptional blockade (Fig. 2g and Supplementary Fig. 6c), suggesting that Piezo1 influences translational efficiency.

To directly quantify translational activity, we performed a puromycin incorporation assay (Fig. 2h).^26^ Yoda1-treated ILC2s presented increased puromycin^+^ translation, with a particular increase in IL-13^+^ translation (Fig. 2i and Supplementary Fig. 6d, e). Furthermore, translation phenotyping confirmed elevated IL-13 protein levels with stable mRNA, whereas the levels of IL-4 and IL-5 remained unchanged (Fig. 2j, k and Supplementary Fig. 6f, g). Collectively, these results demonstrate that Piezo1 activation preferentially promotes IL-13 synthesis by increasing mRNA stability and translational efficiency.

### Piezo1 signaling drives translational reprogramming via Ca^2+^-mTOR axis in ILC2s

**Fig. 3:**
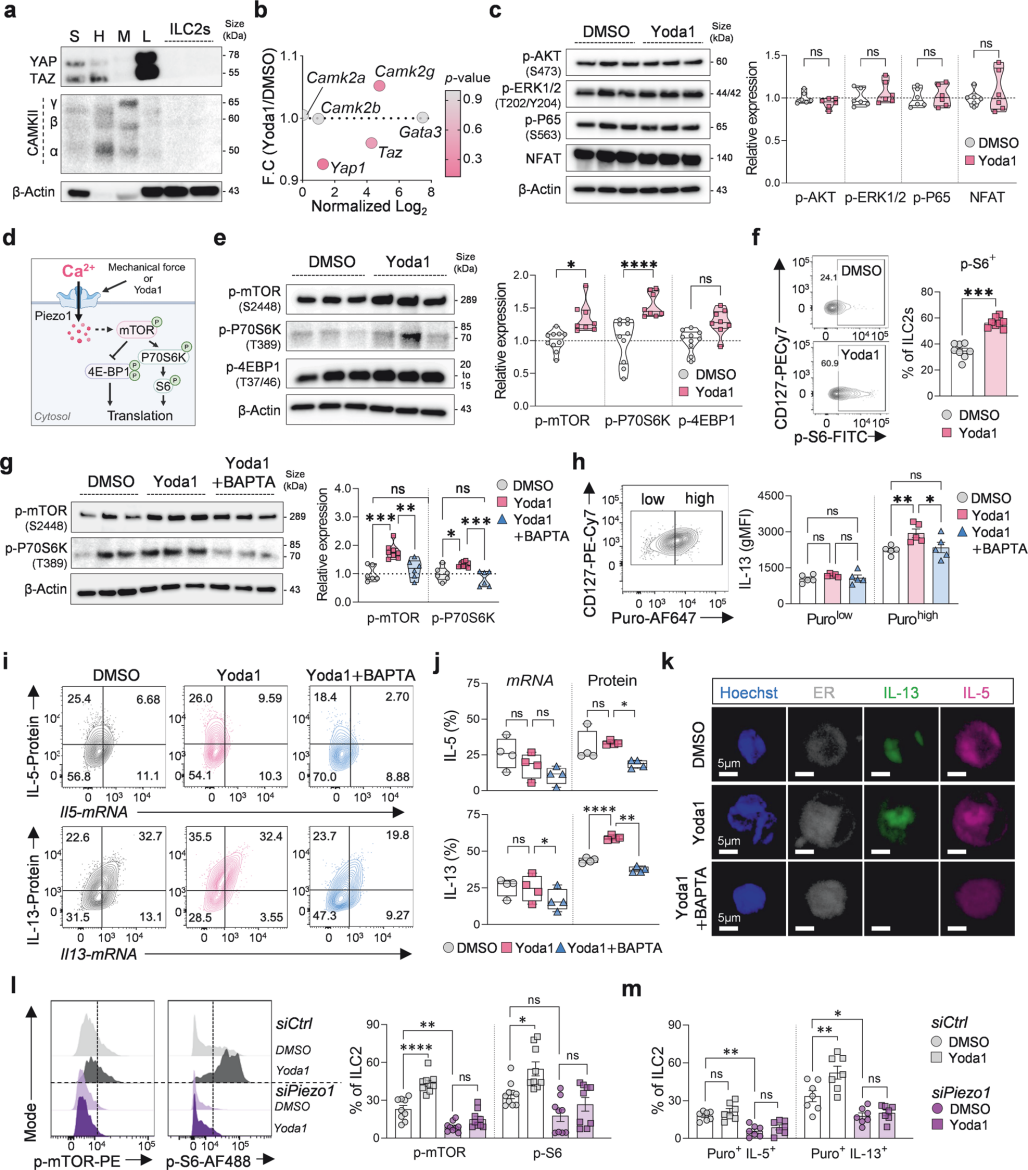
Piezo1 signaling drives translational reprogramming via mTOR in ILC2s. **a** Western blot analysis of YAP/TAZ and CaMKII (α, β, γ) expression in the small intestine (S), heart (H), muscle (M), lung (L), and lung-derived ILC2s. **b** Bulk RNA-seq data showing normalized expression (log₂) of mechanotransduction-related genes (*Camk2a*, *Camk2b*, *Camk2g*, *Yap1*, *Taz*, and *Gata3*) in DMSO- and Yoda1- treated ILC2s. **c** Phosphorylation levels of AKT, ERK1/2, and p65 and protein expression of NFAT in DMSO- or Yoda1-treated ILC2s (*n* = 6). **d** Schematic overview of Piezo1-mediated activation of mTOR signaling. **e** Western blot analysis of phosphorylated mTOR (Ser2448), P70S6K (Thr389), and 4E-BP1 (Thr37/46) after Yoda1 stimulation (*n* = 8–10). **f** Flow cytometry plots and quantification of phosphorylated S6 (p-S6) in DMSO- or Yoda1-treated ILC2s (*n* = 8). **g** Western blot and quantification of p-mTOR and p-P70S6K in DMSO-, Yoda1-, or Yoda1 + BAPTA-treated ILC2s (*n* = 6–8). **h** Flow cytometry analysis of puromycin incorporation in ILC2s under the same treatment conditions (*n* = 5). **i**, **j** Representative PrimeFlow™ cytometry analysis showing the *mRNA* and protein expression of IL-5 (top) and IL-13 (bottom) in DMSO-, Yoda1-, or Yoda1 + BAPTA-treated ILC2s (*n* = 4). **k** Immunofluorescence images of intracellular IL-13 and IL-5 expression in ILC2s (IL-5: magenta, IL-13: green, ER: white, and Hoechst; scale bars = 5 µm). **l** Flow cytometric analysis of p-mTOR and p-S6 in control (*siCtrl*) and Piezo1-knockdown (*siPiezo1*) ILC2s after DMSO or Yoda1 treatment (*n* = 8). **m** Frequencies of puromycin⁺ ILC2s and IL-5⁺ or IL-13⁺ puromycin⁺ ILC2s in control and Piezo1-knockdown ILC2s (*n* = 7). Statistical significance was determined via the Mann–Whitney U test, one-way ANOVA, or two-way ANOVA, as appropriate. The data are presented as the means ± SEMs and are pooled from two to three independent experiments or are representative of similar results. *P < 0.05, **P < 0.01, ***P < 0.001, ****P < 0.0001; ns, not significant

To elucidate the downstream signaling pathways activated by Piezo1 in ILC2s, we examined three key pathways: (1) YAP/TAZ-mediated transcription through mechanosensation,^27,28^ (2) calcium-signaling cascades,^29–31^ and (3) the mammalian target of rapamycin (mTOR) pathway, a central regulator of translation.^32,33^ We observed low YAP/TAZ expression in lung ILC2s and no induction by Piezo1 activation (Fig. 3a, b). Similarly, calcium-activated signaling molecules, including calcium/calmodulin-dependent protein kinase (CaMKII) isoforms (α, β, and γ), exhibited low expression levels in ILC2s (Fig. 3a, b). Furthermore, Yoda1 treatment did not alter the phosphorylation of AKT, ERK1/2, or P65 or the expression of NFAT (Fig. 3c and Supplementary Fig. 7a), suggesting the limited involvement of classical calcium-dependent pathways. In contrast, Piezo1 activation by Yoda1 significantly induced the phosphorylation of mTOR and its downstream target P70S6K, whereas the phosphorylation of 4E-BP1 remained unchanged (Fig. 3d, e). Increased phosphorylation of the ribosomal protein S6 was also observed, reflecting enhanced translational capacity (Fig. 3f). This finding was further supported by time-course analyses showing rapid induction of mTOR, P70S6K, and S6 phosphorylation following Yoda1 treatment (Supplementary Fig. 7b, c).

To test whether these effects were calcium dependent, we depleted calcium via BAPTA. This led to a significant reduction in Yoda1-induced phosphorylation of mTOR and P70S6K (Fig. 3g) as well as p-S6 levels (Supplementary Fig. 7d), indicating that calcium influx is essential for Piezo1–mTOR signaling. Consistently, the number of puromycin⁺ IL-13⁺ ILC2s was reduced under calcium depletion, suggesting impaired translational activity (Fig. 3h and Supplementary Fig. 7e, f). Similarly, treatment with the mTOR inhibitor rapamycin reduced Yoda1-induced IL-13 translation (Supplementary Fig. 7d, e). The PrimeFlow™ assay further confirmed that Yoda1 increased IL-13 protein levels without altering *Il13* mRNA expression, which was consistent with translational regulation, whereas the levels of the IL-4 and IL-5 proteins and transcripts remained unaffected (Fig. 3i, j and Supplementary Fig. 7g). Confocal imaging revealed the accumulation of IL-13 within the ER compartment upon Yoda1 stimulation, which was abolished by BAPTA, whereas the level of IL-5 was unchanged (Fig. 3k). These findings were reproducible with other calcium chelators and with rapamycin (Supplementary Fig. 8a–d).

Finally, *siRNA*-mediated knockdown of Piezo1 further demonstrated its requirement for mTOR phosphorylation and translational reprogramming. Piezo1-deficient ILC2s presented a marked reduction in Yoda1-induced phosphorylation of mTOR and S6 and a reduced number of puromycin-positive translating ILC2s (Fig. 3l, m and Supplementary Fig. 9a, b). Additionally, using defined-stiffness PDMS hydrogels to simulate mechanical stress, we confirmed that stiffness-induced mTOR activation and translational activity were also Piezo1 dependent (Supplementary Fig. 9c, d). Collectively, these results demonstrate that Piezo1–Ca²⁺–mTOR signaling enhances translational activity in ILC2s, selectively promotes IL-13 production, and shapes ILC2 effector function under mechanical stimulation.

### Piezo1 deletion impairs ILC2 functionality through translation-related pathways

**Fig. 4:**
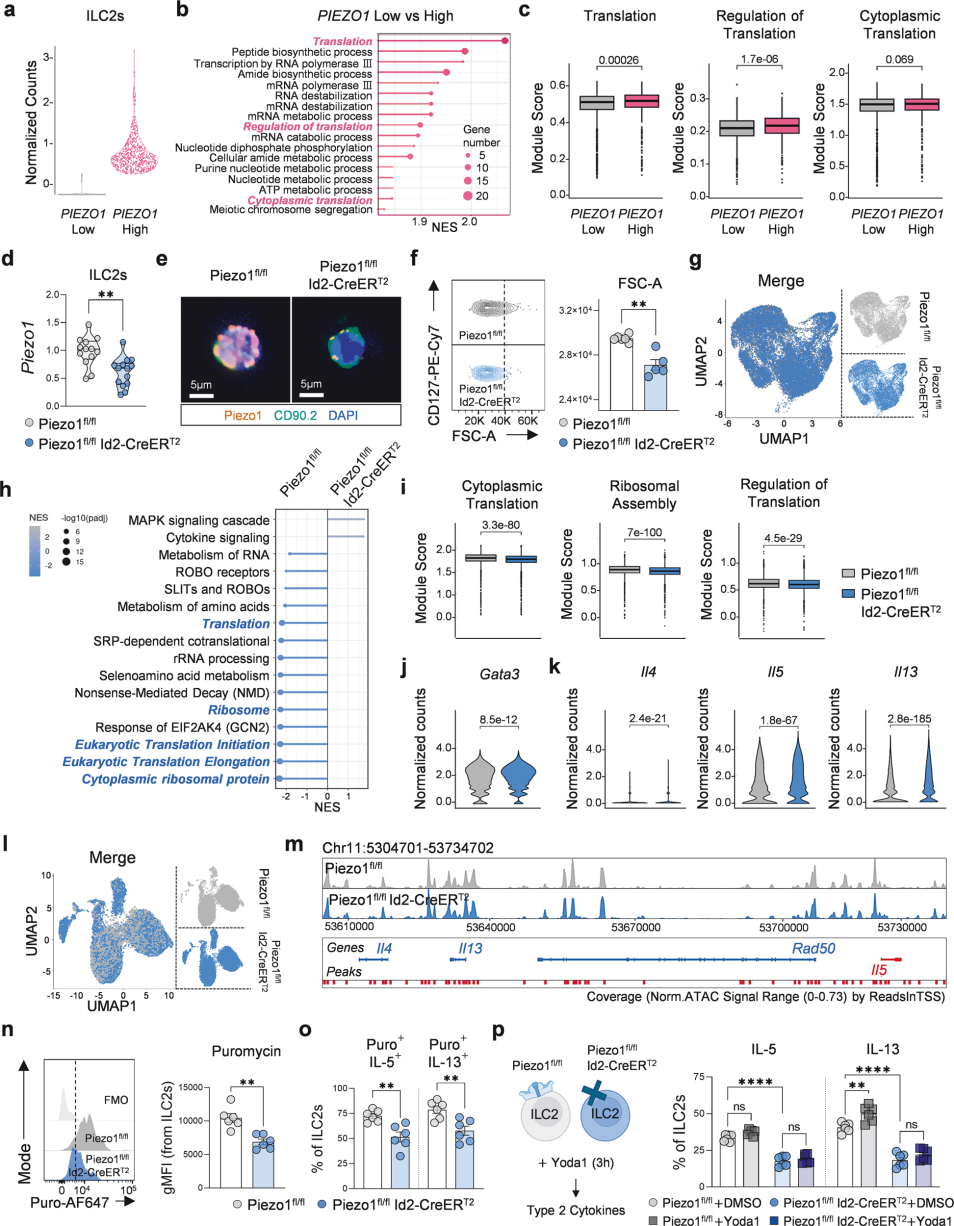
Piezo1 deletion impairs ILC2 functionality and translation activity. (**a**–**c**) scRNA-seq analysis of human lung ILC2s stratified by *PIEZO1* expression. **a** Violin plot showing the distribution of *PIEZO1* expression. **b** GSEA identifying enrichment of translation-related pathways in *PIEZO1*^high^ vs. *PIEZO1*^low^ ILC2s. **c** Module scores for translation-associated gene sets. **d** qPCR analysis of *Piezo1* mRNA levels in lung ILC2s from Piezo1^fl/fl^ and Piezo1^fl/fl^ Id2-CreER^T2^ mice (*n* = 13–14). **e** Representative immunofluorescence images showing Piezo1 and CD90.2 expression in lung ILC2s. (Piezo1: orange, CD90.2: green, and DAPI, scale bars = 5 µm), **f** Representative flow cytometry plots and quantification of the forward scatter area (FSC-A) in ILC2s (*n* = 5). **g** UMAP projection of scRNA-seq profiles comparing Piezo1^fl/fl^ (gray) and Piezo1^fl/fl^ Id2-CreER^T2^ (blue) ILC2s. GSEA (**h**) and module score analysis (**i**) revealing reduced translation and ribosomal biogenesis in *Piezo1*-deficient ILC2s. **j**, **k** Violin plots showing normalized gene expression of *Gata3, Il4, Il5*, and *Il13* in Piezo1^fl/fl^ and Piezo1^fl/fl^ Id2-CreER^T2^ ILC2s from scRNA-seq. **l** UMAP projection of scATAC-seq profiles from Piezo1^fl/fl^ and Piezo1^fl/fl^ Id2-CreER^T2^ ILC2s. **m** Chromatin accessibility tracks at the *Il4, Il5, Il13*, and *Rad50* loci in Piezo1^fl/fl^ (gray) and Piezo1^fl/fl^ Id2-CreER^T2^ (blue) ILC2s. **n**, **o** Flow cytometry analysis showing the frequencies of puromycin⁺ ILC2s (**n**) and IL-5⁺- and IL-13⁺- ILC2s (**o**) in Piezo1^fl/fl^ and Piezo1^fl/fl^ Id2-CreER^T2^ mice (*n* = 6). **p** Schematic and flow cytometry plots showing IL-5⁺ and IL-13⁺ ILC2s in Piezo1^fl/fl^ and Piezo1^fl/fl^ Id2-CreER^T2^ mice following Yoda1 or DMSO vehicle treatment (*n* = 6). Statistical significance was determined via the Mann–Whitney U test, one-way ANOVA, or two-way ANOVA. The data are presented as the means ± SEMs and are representative of or pooled from two or more independent experiments. **P < 0.01, ****P < 0.0001; ns, not significant

To further clarify the role of Piezo1 in ILC2 functionality, we first analyzed public datasets from human lung-resident ILC2s.^22^ Stratification into high- and low-*PIEZO1*-expressing groups revealed that high *PIEZO1* expression was associated with enriched gene sets related to translation regultion, peptide biosynthesis, and cytoplasmic translation (Fig. 4a–c). These findings are consistent with our earlier results (Fig. 2c), supporting a model in which Piezo1 activation enhances the translational capacity of ILC2s. To assess the impact of Piezo1 deficiency directly, we generated tamoxifen-inducible Piezo1- conditional knockout mice (Piezo1^fl/fl^ Id2-CreER^T2^, cKO) and administered tamoxifen to delete Piezo1 in ILC2s (Supplementary Fig. 10a). We confirmed efficient deletion at both the mRNA and protein levels in lung ILC2s (Fig. 4d, e and Supplementary Fig. 10c), with no reduction observed in other Id2⁺ cells, such as T cells, B cells, or macrophages (Supplementary Fig. 10b). Piezo1-deficient ILC2s presented normal basal Ca^2+^ levels and apoptosis but displayed reduced cell size and impaired proliferation capacity (Fig. 4f and Supplementary Fig. 10d–g). Given the role of Id2 in the development of multiple immune lineages,^34,35^ we assessed off-target effects on other immune subsets. Piezo1 cKO mice presented a modest reduction in ILC2 frequencies in the bone marrow and lung (Supplementary Fig. 11a, c) and slight reductions in splenic NK cells and lung CD103⁺ cDC1 cells (Supplementary Fig. 11b, d–f).

To investigate how Piezo1 regulates ILC2 function at the molecular level, we performed single-cell RNA sequencing (scRNA-seq) and single-cell ATAC sequencing (scATAC-seq) on lung ILC2s from Piezo1 cKO mice and littermate controls. scRNA-seq analysis revealed the downregulation of gene programs related to translation and cytoplasmic ribosomal protein synthesis (Fig. 4g–i). In addition, genes involved in actin cytoskeleton reorganization, such as those in the cdc42 GTPase pathway,^36^ were slightly downregulated (Supplementary Fig. 12a–c). However, key transcription factors, including *Gata3* and *Id2*, as well as type 2 cytokines (*Il4*, *Il5*, and *Il13*), and *Rad50* remained unaffected (Fig. 4j, k and Supplementary Fig. 12d). Consistently, scATAC-seq data revealed no substantial changes in chromatin accessibility at these gene promoters (Fig. 4l, m and Supplementary Fig. 12e–g). To functionally validate these findings, we assessed protein translation and cytokine production in Piezo1-deficient ILC2s. Piezo1 cKO ILC2s, including both the IL-13⁺ and IL-5⁺ populations, presented reduced frequencies of puromycin⁺-transformed cells (Fig. 4n, o and Supplementary Fig. 12h). Upon Yoda1 stimulation, however, only IL-13 translation was enhanced in control ILC2s, a response that was absent in Piezo1-deficient cells (Fig. 4p). These results suggest that Piezo1 deletion limits the translational capacity of ILCs without altering their transcriptional or epigenetic state.

### Conditional deletion of Piezo1 in ILC2s attenuates acute lung inflammation and chronic lung fibrosis

**Fig. 5:**
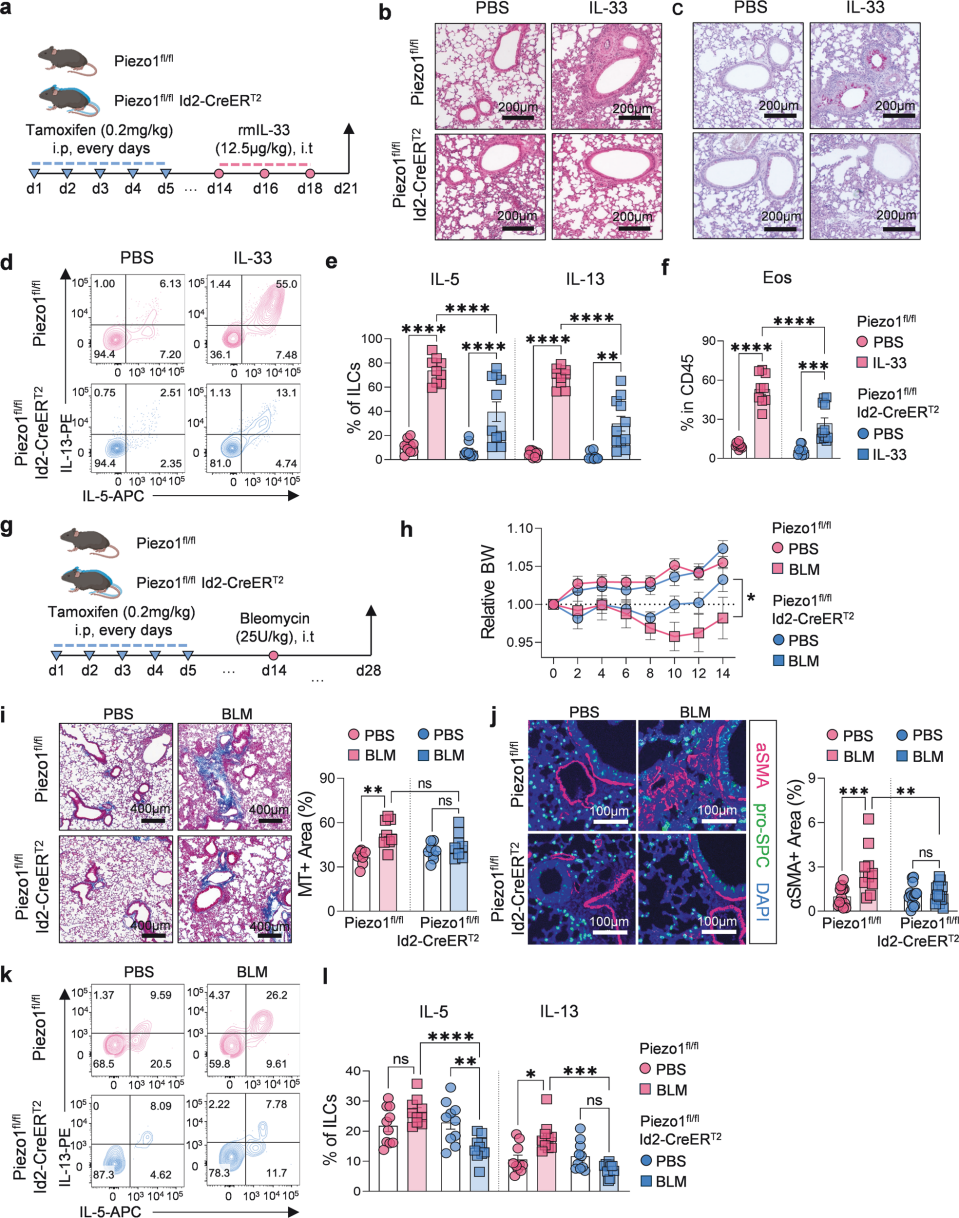
Conditional deletion of Piezo1 in ILC2s reduces acute airway inflammation and chronic lung fibrosis. **a** Schematic of the tamoxifen-inducible Piezo1^fl/fl^ and Piezo1^fl/fl^ Id2-CreER^T2^ (cKO) mouse model and the IL-33–induced acute lung inflammation protocol. Representative H&E (**b**) and PAS (**c**) images of lung sections from Piezo1^fl/fl^ and cKO mice following IL-33 treatment. (scale bars = 200 µm), **d** Representative flow cytometry plots of IL-5⁺ and IL-13⁺ lung ILC2s. **e** Quantification of IL-5⁺ and IL-13⁺ ILC2s (*n* = 9–11). **f** Frequencies of lung eosinophils after IL-33 administration (n = 9–11). **g** Schematic of the bleomycin (BLM)-induced lung fibrosis model in Piezo1^fl/fl^ and cKO mice. **h** Body weight changes over the course of fibrosis (*n* = 9–12). **i** Representative Masson’s trichrome (MT) staining and quantification of the fibrotic area in lung tissue (*n* = 7–8) (scale bars = 400 µm). **j** Representative image of α-SMA immunofluorescence staining (magenta) costained with pro-SPC (green) and DAPI (blue) (scale bars = 100 µm) and quantification of the α-SMA⁺ area (*n* = 10–14). Representative flow cytometry plots (**k**) and quantification (**l**) of IL-5⁺ and IL-13⁺ ILC2s in lung tissue from Piezo1^fl/fl^ and cKO mice following BLM exposure (*n* = 11–13). Statistical significance was determined via one-way ANOVA or two-way ANOVA, as appropriate. The data are presented as the means ± SEMs and were pooled from at least two independent experiments. *P < 0.05, **P < 0.01, ***P < 0.001, ****P < 0.0001; ns, not significant

To investigate the role of Piezo1 in ILC2-mediated allergic inflammation and fibrosis, we utilized two physiologically relevant mouse models that are known to increase mechanical stress within the lung microenvironment: an acute IL-33-induced acute allergic airway inflammation model^37,38^ and a chronic bleomycin (BLM)-induced lung fibrosis model (Fig. 5a, g).^39,40^ In the IL-33 model, intratracheal administration of IL-33 induces rapid activation and expansion of ILC2s, leading to eosinophilic inflammation and mucus hypersecretion. Piezo1 cKO mice presented significantly reduced airway inflammation and mucus production, as shown by H&E and PAS staining (Fig. 5b, c). ILC2 frequencies were decreased (Supplementary Fig. 13a), along with reductions in IL-5 and IL-13 production (Fig. 5d, e) and eosinophil infiltration (Fig. 5f and Supplementary Fig. 13b). The other major immune populations were not significantly affected (Supplementary Fig. 13c, d). Consistent phenotypes were observed in the *Alternaria alternata* model (Supplementary Fig. 14a–f), further supporting the role of Piezo1 in promoting type 2 inflammation.

In the BLM model, repeated administration of bleomycin leads to progressive fibrosis driven in part by ILC2-derived IL-13, which promotes fibroblast activation and extracellular matrix deposition.^39,40^ Piezo1 cKO mice showed accelerated recovery from weight loss (Fig. 5h) and markedly reduced fibrotic areas, as shown by Masson’s trichrome staining (Fig. 5i) and α-SMA immunofluorescence (Fig. 5j). The expression of fibrosis-related genes such as *Acta2, Col3a1*, and *Mmp12* was also significantly downregulated (Supplementary Fig. 15a). In line with these observations, the frequencies of IL-5⁺ and IL-13⁺ ILC2s were reduced (Fig. 5k, l), whereas other immune cell populations, including eosinophils, were only modestly affected (Supplementary Fig. 15b–f).

Collectively, these results demonstrate that Piezo1 functions as a key mechanosensor in ILC2s, amplifying cytokine production in response to tissue remodeling–associated mechanical cues and contributing to both acute allergic inflammation and chronic lung fibrosis.

### Pharmacological mTOR inhibition suppresses ILC2-driven lung inflammation and fibrosis

**Fig. 6:**
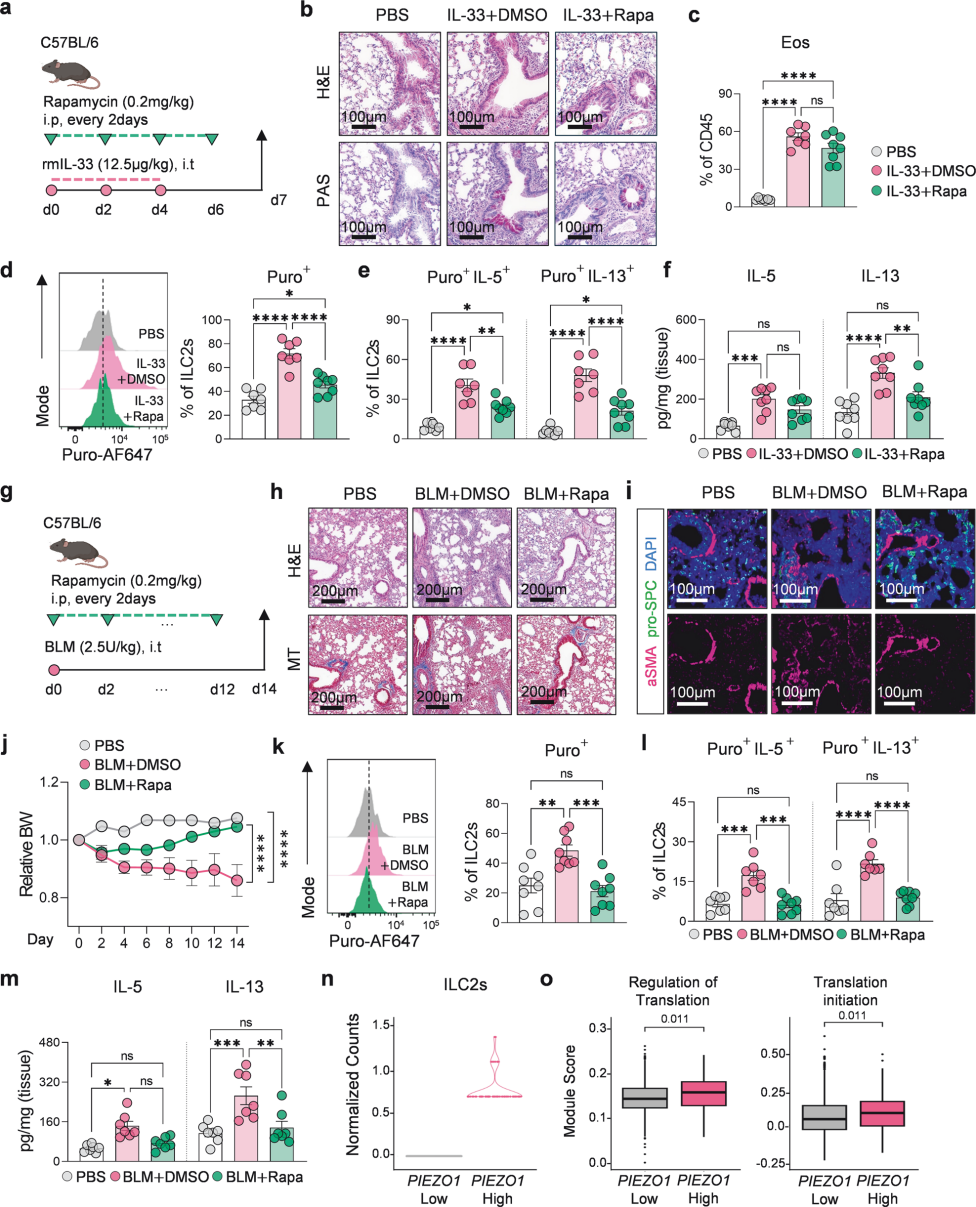
mTOR inhibition by rapamycin suppresses ILC2 translation and attenuates lung inflammation and fibrosis. **a** Schematic of the IL-33–induced airway inflammation model with intraperitoneal rapamycin administration (0.2 mg/kg, every 2 days). **b** Representative H&E and PAS staining of lung tissues from PBS-, IL-33–, and IL-33 + rapamycin (Rapa)-treated mice. (scale bars = 100 µm). **c** Frequencies of eosinophils among CD45⁺ lung cells (*n* = 7–8). **d** Representative histograms and quantification of puromycin⁺ (translating) ILC2s (*n* = 7–8). **e** Frequencies of IL-5⁺ and IL-13⁺ puromycin⁺ ILC2s (*n* = 7–8). **f** Concentrations of IL-5 and IL-13 in lung homogenates measured by ELISA (*n* = 8). **g** Schematic of the bleomycin (BLM)-induced lung fibrosis model with rapamycin treatment. **h** Representative H&E- and Masson’s trichrome-stained lung sections showing fibrotic areas. (scale bars = 200 µm). **i** Representative α-SMA immunofluorescence staining (magenta) costained with pro-SPC (green) and DAPI (blue) (scale bars = 100 µm). **j** Time course of relative body weight changes following BLM and rapamycin treatment (*n* = 8). **k** Representative histograms and quantification of puromycin⁺ ILC2s in fibrotic lungs (*n* = 8). **l** Frequencies of IL-5⁺ and IL-13⁺ puromycin⁺ ILC2s (*n* = 7–8). **m** Concentrations of IL-5 and IL-13 in lung homogenates from BLM-treated mice (*n* = 7). Public mouse lung scRNA-seq datasets showing that *Piezo1*^high^ ILC2s exhibit elevated translation-associated gene expression (**n**) and higher module scores for translation-related pathways (**o**) than docompared to *Piezo1*^low^ ILC2s. Statistical significance was determined via one-way ANOVA or two-way ANOVA, as appropriate. The data are presented as the means ± SEMs and were pooled from at least two independent experiments. *P < 0.05, **P < 0.01, ***P < 0.001, ****P < 0.0001; ns, not significant

On the basis of our findings that Piezo1 promotes IL-13 production through mTOR signaling in ILC2s, we tested whether pharmacological inhibition of mTOR could therapeutically suppress ILC2-mediated lung pathology. As direct in vivo targeting of Piezo1 remains challenging, we focused on its downstream effector pathway via the use of rapamycin, an FDA-approved mTOR inhibitor. Rapamycin was administered intraperitoneally during disease induction in both the IL-33–induced acute airway inflammation and bleomycin (BLM)-induced chronic fibrosis models (Fig. 6a, g). In the IL-33 model, rapamycin treatment significantly reduced airway inflammation and mucus production (Fig. 6b) and decreased eosinophil infiltration (Fig. 6c). Importantly, it also led to a reduction in the frequency of puromycin⁺ actively translating ILC2s (Fig. 6d) and IL-5⁺ and IL-13⁺ translating ILC2s (Fig. 6e). ELISA confirmed a marked decrease in tissue IL-13 and a moderate reduction in IL-5 levels (Fig. 6f).

Similar therapeutic effects were observed in the chronic fibrosis model. Rapamycin-treated mice presented attenuated fibrotic remodeling, with reduced α-SMA⁺ myofibroblast accumulation (Fig. 6h, i), downregulation of fibrosis-related genes (Supplementary Fig. 16a), and improved weight recovery (Fig. 6j). The levels of translationally active and cytokine-producing ILC2s were also significantly reduced (Fig. 6k, l), as was the level of the IL-13 protein in lung homogenates (Fig. 6m). Flow cytometric analysis further confirmed that ILC2s were the predominant source of IL-13 in both models, whereas CD4⁺ T cells contributed minimally (Supplementary Fig. 16b–d). Finally, scRNA-seq analysis of lung ILC2s from a murine model of spontaneous fibrosis^41^ revealed that *Piezo1*-high ILC2s presented increased module scores for translation-related pathways compared with their *Piezo1*-low counterparts (Fig. 6n, o). These results underscore the relevance of Piezo1–mTOR signaling in disease pathogenesis and highlight mTOR as a tractable therapeutic target for modulating mechanosensitive immune responses in type 2 lung inflammation and fibrosis.

## Discussion

The lungs harbor resident ILC2s that patrol barrier tissues and rapidly initiate immune responses to environmental insults, such as pathogens and pollutants.^42^ While ILC2s are essential for maintaining lung homeostasis, their excessive activation drives acute and chronic inflammation, ultimately leading to tissue remodeling and fibrosis.^15,43^ Notably, the lung is constantly exposed to mechanical forces—including airflow, cyclic stretch, and pressure changes—that can compromise structural integrity and exacerbate immune-mediated pathology.^2,10,44^ Although the critical role of ILC2s in lung immunity is well established, the impact of mechanical cues on ILC2 function, particularly in inflamed tissues, remains poorly understood.

In this study, we identified Piezo1, a mechanosensitive ion channel, as a key regulator of cytokine production in both murine and human lung-resident ILC2s. Activation of Piezo1 enhances IL-13-biased translation by promoting *Il13* mRNA translation without altering the transcription of *Il13* or other type 2 cytokines. These findings reveal a novel post-transcriptional checkpoint involved in ILC2 effector programming. In support of this, gene set enrichment analysis (GSEA) of human lung ILC2s revealed that high *PIEZO1* expression is correlated with increased translation efficiency and mRNA stability—two critical regulators of cytokine output.^45,46^ Furthermore, scRNA-seq and scATAC-seq data indicated that Piezo1 deficiency did not significantly alter transcripts levels or chromatin accessibility of type 2 cytokine gene in mice ILC2a, supporting the conclusion that Piezo1 functions primarily at the translational level. Mechanistically, Piezo1 activated the Ca^2+^-mTOR signaling pathway, a critical regulator of protein synthesis,^47^ to drive IL-13-biased production. This mechanism is consistent with previous work in intestinal ILC3s, where Piezo1-mTOR signaling promotes IL-17A production,^48^ suggesting a broader, context-dependent role for Piezo1 in modulating effector functions across ILC subsets. To evaluate the physiological relevance of this pathway, we utilized Piezo1^fl/fl^ Id2-CreER^T2^ mice to achieve inducible deletion of Piezo1 in mature ILC2s. At steady state, Piezo1-deficient ILC2s presented no apparent developmental or phenotypic abnormalities. However, under disease conditions, Piezo1 deletion results in significantly impaired production of cytokines, including IL-13, leading to reduced airway inflammation and fibrosis. These results underscore the importance of Piezo1-mediated mechanotransduction in enabling ILC2s to mount effective pathogenic responses in the inflamed lung.

To our knowledge, only a few studies have investigated Piezo1 function in ILC2s, with notably divergent results. For example, Kim et al. demonstrated that mechanical stretching of the stomach, induced by a chitin-rich diet or air inflation, increased IL-5 production in stomach-resident ILC2s through a Piezo1–tuft cell–IL-25 axis.^49^ These findings suggest that Piezo1 may regulate ILC2 activation via interactions with structural cells, a mechanism that may also operate in the lung microenvironment. In contrast, Hurrell et al. reported constitutive Piezo1 deletion. Piezo1 in Il7r-expressing cells exacerbated IL-33-induced airway hyperresponsiveness, suggesting a protective role for Piezo1.^50^ In contrast, our study employed an inducible deletion model targeting mature ILC2s and revealed a cell-intrinsic role for Piezo1 in enhancing mTOR-dependent translation and effector function. These differing outcomes may be attributed to variations in genetic strategies (constitutive vs. inducible), developmental timing of gene deletion (progenitor vs. mature stage), and disease models (airway hyperreactivity vs. acute/chronic inflammation). Notably, our findings align with those of Liu et al., who also demonstrated that Piezo1 activation in intestinal ILC3s promotes IL-17A via mTOR signaling.^48^ Collectively, these studies highlight the context-specific and subset-specific roles of Piezo1 in innate lymphoid cell biology.

In addition to its role in translational regulation, the mechanism of Piezo1 activation in ILC2s under pathological conditions is a key consideration. In diseases such as allergic inflammation, fibrosis, or environmental injury, extracellular matrix remodeling and tissue stiffening increase mechanical forces and airway pressure—factors that can directly activate Piezo1.^51^ While Piezo1 is recognized primarily as a mechanosensor, recent studies have shown that it can also be sensitized by inflammatory mediators such as lipid metabolites and cytokines, lowering its activation threshold and enhancing responsiveness.^52–54^ This dual sensitivity allows Piezo1 to detect subtle mechanical cues and amplify downstream signals in inflamed tissues. Using a pressure-based mechanical stimulation system, we confirmed that ILC2s can sense physical forces via Piezo1, although modest IL-13 induction suggests that full activation in vivo may require additional inflammatory inputs. Complementary experiments with Yoda1, a selective Piezo1 agonist, revealed Piezo1-specific effects and consistently enhanced IL-13 production. Notably, Piezo1 activation selectively increased IL-13 without significantly affecting IL-4 or IL-5, indicating a nonredundant role in modulating IL-13–driven ILC2 functions. Thus, Piezo1 serves as an integrative sensor of biomechanical and immunological inputs to fine-tune ILC2 responses. In addition to its role in the lung, Piezo1 has been linked to immune regulation in adipose tissue inflammation, suggesting that it may contribute to type 2 inflammation in multiple tissues.^53^ These findings may help explain the increased severity of Th2-associated diseases such as asthma and atopic dermatitis in obese individuals with chronic adipose inflammation. Despite the strengths of our study, certain limitations remain. Although our conditional Piezo1 knockout model minimizes developmental effects, deletion in Id2-expressing cells may not be fully restricted to ILC2s.^34^ However, Piezo1 expression was minimal in other Id2^+^ immune subsets, suggesting limited off-target effects. We also used GsMTx4 as a functional Piezo1 inhibitor, but given its activity on other mechanosensitive channels, including TRPs,^55^ the results were interpreted alongside genetic models (Piezo1 *si*RNA and conditional knockout) to ensure specificity. Furthermore, discrepancies between in vitro and in vivo findings suggest that Piezo1 function is shaped by the context and duration of activation. Transient stimulation primarily enhanced IL-13 translation, whereas sustained in vivo activation also influenced IL-5 production. These results underscore the need for further studies to define the temporal and contextual regulation of Piezo1 signaling in ILC2s under disease conditions.

In conclusion, our findings identify Piezo1 as a pivotal modulator of ILC2 pathogenicity by promoting IL-13-biased translation through the Ca²⁺–mTOR axis. This mechanotransduction pathway enables ILC2s to respond to dynamic tissue environments shaped by inflammation and mechanical stress. Given the challenge of directly targeting Piezo1 due to its broad expression and lack of specific inhibitors, our data support the feasibility of therapeutically modulating downstream translational programs. Pharmacologic inhibition of mTOR with rapamycin effectively dampened IL-13 production and attenuated lung inflammation and fibrosis in vivo, establishing a proof-of-concept for targeting the Piezo1–mTOR axis in type 2 lung diseases (Supplementary Fig. 17). These results provide a mechanistic framework for future therapeutic strategies that exploit mechanotransduction in immune cells to control chronic inflammatory disorders.

## Materials and methods

### Mice

Both male and female wild-type C57BL/6 J (WT) mice, 6–10 weeks old, were purchased from Koatech (Gyeonggi-do, South Korea) and used in all in vivo and in vitro experiments. No sex-dependent differences were observed, and data from both sexes were pooled for analysis unless otherwise indicated. Piezo1 conditional knockout (cKO) mice were generated by breeding Piezo1^flox/flox^ (stock no. 029213) with Id2-creER^T2^ (stock no. 016222) mice from Jackson Laboratories (Bar Harbor, ME, USA). Cre translocation was induced by administering 100 mg/kg tamoxifen (Sigma, T5648) intraperitoneally every day for 5 days. The mice were housed in specific pathogen-free conditions at Seoul National University Hospital (SNUH). All procedures followed the guidelines of the Institutional Animal Care and Use Committee (IACUC #20-0099-S1A0 and #23-0038-C1A0) at SNUH, accredited by AAALAC International.

### Mouse single-cell preparation

Whole lungs and visceral white adipose tissue were collected from euthanized mice. For lung tissue, the lungs were minced and digested in 1 mg/mL collagenase type 4 (Worthington, LS004189) and 5 µg/mL DNase I (Sigma‒Aldrich, DN25) in RPMI 1640 medium (Biowest, L0500) at 37 °C for 1.5 hours. For visceral white adipose tissue, the tissue was minced, washed with 1X PBS, and centrifuged to remove debris before being digested in the same enzyme mixture at 37 °C for 45 minutes. The resulting cell suspensions from all the tissues were filtered through a 40 µm strainer, treated with RBC lysis buffer (BioLegend, 420301), and resuspended in buffer for analysis.

### Mouse ILC2 culture and Piezo1 stimulation

Murine lung ILC2s were enriched from recombinant mouse (rm) IL-33 (BioLegend, 580508)-treated mice via the EasySep Mouse Pan-ILC Enrichment Kit (Stem Cell, 19875) and CD45 microbeads (Miltenyi Biotec, 130–052–301). The cells were cultured in RPMI 1640 with 10% FBS, 10 µg/mL gentamicin (Biowest, L0011-010), rmIL-2 (BioLegend, 575406), rmIL-7 (BioLegend, 577806), and rmIL-33 (10 ng/mL each) at 37 °C. For alarmin stimulation, ILC2s were treated with rmIL-25 (BioLegend, 587304), rmIL-33, or rmTSLP (R&D Systems, 555-TS-010/CF) in media containing rmIL-2 or rmIL-7 (10 ng/mL each) for 10 days. ILC2s from tamoxifen-inducible Piezo1^fl/fl^ and Piezo1^fl/fl^ Id2-CreER^T2^ mice were sorted via a FACS Aria III sorter (BD Biosciences). For Piezo1 modulation, ILC2s were treated with 5 µM Yoda1 (Tocris, 5586), 1–1000 μM GsMTx4 (R&D Systems, 4912 and MCE, HY-P1410), 2 mM calcium chelators (BAPTA, Cayman) or 100 µM rapamycin (Sigma, 553210) for the designated time points.

### Whole-cell patch clamp for Piezo1 activation

Whole-cell patch-clamp recordings were performed via an Axopatch-200B amplifier and pCLAMP software. Piezo1 activation was induced with 5 µM Yoda1 (Tocris, 5586), and membrane currents were recorded. The bath solution contained 145 mM NaCl, 3.6 mM KCl, 10 mM HEPES, 5 mM glucose, 1 mM MgCl_2_, and 1.3 mM CaCl_2_ (pH 7.4).

### Intracellular Ca^2+^ measurement

For intracellular Ca^2+^ measurement, ILC2s were loaded with CAL-520 AM (AAT Bioquest, 21130) to monitor Ca^2+^ flux. The cells were placed in an 8-well Nunc® Lab-Tek® II chamber (Thermo Fisher Scientific, 177445) coated with poly-L-lysine (Sigma, P4707) and stimulated. Live-cell imaging was performed on a Nikon A1 confocal microscope, and the fluorescence intensity was monitored every second via a 488 nm excitation laser. The change in fluorescence intensity was tracked at ~1 frame/s within the 500–550 nm detection range, with or without 2 mM BAPTA in HHBS.

### siRNA-mediated gene knockdown

For *Piezo1* knockdown in murine lung ILC2s, 1 µM Accell SMARTpool Mouse *Piezo1* siRNA (Horizon Discovery, E–061455–00–0010, 234839) or 1 µM Dharmacon Accell Nontargeting Pool (Horizon Discovery, D–001910–10–05) was added to Accell siRNA delivery media (Horizon Discovery, B–005000–100) with rmIL-2,7,33 (10 ng/mL each) and at least 2.5% FBS. The delivery of siRNAs was validated after 72 hours by qPCR, CAL-520 AM, and immunofluorescence imaging, and cytokine levels were determined by flow cytometry.

### Human blood sample collection

Peripheral blood samples were obtained from 12 healthy controls who provided written informed consent. The study protocol was approved by the Seoul National University Hospital Institutional Review Board (approval number: 2302–005–1400) and followed the guidelines of the World Medical Association’s Declaration of Helsinki. Human ILC2s were enriched from PBMCs using Ficoll-Paque density gradient solution and via the Easysep Human Pan-ILC Enrichment Kit (Stem Cell, 17975). The cells were cultured in RPMI 1640 with 10% FBS, 10 µg/mL gentamicin, recombinant human (rh) IL-2 (BioLegend, 589106), rhIL-7 (BioLegend, 581906), and rhIL-33 (BioLegend, 581806) (30 ng/mL each) at 37 °C. For Piezo1 modulation, ILC2s were treated with 5 µM Yoda1 for the designated time points.

### Flow cytometry analysis

The isolated single cells were stained with the Zombie Aqua Fixable Viability Kit (BioLegend, 423102) and blocked with an anti-CD16/32 FC blocking antibody (BioLegend, 156604). The surface markers were stained with specific antibodies, and intracellular cytokine staining was performed on cells stimulated with DMSO (0.1%) or Yoda1 (5 µM), or 100 ng/ml PMA (Sigma, P8139-1MG) + 1 µg/ml ionomycin (Sigma, I0364-1MG) followed by incubation with GolgiStop™ (BD Biosciences, 554724). After surface staining, the cells were fixed and permeabilized via the Cytofix/Cytoperm Kit (BD Biosciences, 554715) or the Foxp3/Transcription Factor Staining Buffer Set (Invitrogen, 00–5523–00) and then stained with specific antibodies. Cytokine mRNA expression was detected via the PrimeFlow™ assay via standard mouse probes for *Il4*, *Il5*, and *Il13*, following the manufacturer’s instructions. For phosphoprotein detection, surface-stained cells were fixed with fixation buffer (BioLegend, 420801), permeabilized with True-Phos Perm Buffer (BioLegend, 425401) and stained with relevant antibodies. Flow cytometry data were analyzed via FlowJo software v10 (BD, NJ, USA) with BD LSR Fortessa™ X-20 and BD LSRII^TM^ (BD, NJ, USA) instruments.

### Cyclic air pressure chamber (pressure-controlled desiccator)

The custom-built cyclic air pressure chamber was designed via Inventor 2020 (Autodesk) and manufactured from transparent acrylic. It includes two DAP-3657B DC24V air pumps (Motorbank), a solenoid valve VDW213 (Aonetech), and an XGZP6847-040KPGPN pressure sensor (CFSensor). Pressure regulation was controlled via a Raspberry Pi 3 microprocessor. The chamber operated at 1 atm ± 10 kPa, simulating murine respiratory conditions at a frequency of 1–2 Hz. The airflow was maintained at physiological temperature (37 °C).

### PDMS hydrogels

To prepare the defined stiffness PDMS hydrogel, part A and part B components of Dow Corning Sylgard 527 silicone dielectric gel (Ellsworth Adhesives, 1696742) were mixed at the following ratios to obtain the appropriate tensions by Lee et al.^19^ For the 2 kPa gel, the gel ratio of A:B was 6:5, and for the 50 kPa gel, the ratio of A:B was 3:10. The plates were coated with the hydrogel and incubated overnight at 60 °C. For the 96-well plates, 50 µl of gel was coated per well. After solidification, the hydrogel-coated plates were sterilized by ultraviolet irradiation and coated with 5 µg/ml rat-tail type 1 collagen (Corning, 354236) and 1 µg/ml bovine fibronectin (Sigma, F1141-1 mg) in PBS overnight at 37 °C, followed by washing with PBS.

### Western blotting

Cultured ILC2s were lysed in RIPA buffer (Biosesang, RC2002-050-00) containing protease (Sigma, P1860-1 ML) and phosphatase inhibitors (GenDePOT, P3100-001). The protein concentration was quantified via the Pierce™ BCA Protein Assay Kit (Thermo Fisher Scientific, 23227). The lysates were separated on 4–20% gradient gels (Bio-Rad, 4561096), transferred to Immobilon PVDF membranes (Merck Millipore, IPVH00010), and immunoblotted with specific antibodies. Images were acquired with an Amersham Imager 680 (Cytiva, USA), and densitometric analysis was performed via ImageJ (NIH, USA).

### ELISA

Cytokine levels (IL-4, IL-5, and IL-13) in mouse ILC2 culture supernatants and lung homogenates were measured via ELISA kits (R&D Systems, DY404-05, DY405-05, and DY413-05). Standard curves were generated to quantify the cytokine concentrations.

### Quantitative real-time PCR

Total RNA was extracted via TRIzol (Invitrogen, 15596018) and synthesized into cDNA via the SeniFAST cDNA synthesis kit (Bioline, 1708891). qPCR was performed with the SeniFAST SYBR Lo-ROX kit (Bioline, BIO-94020) via a CFX96 Real-Time PCR Detection System (Bio-Rad, USA). Gene expression levels were normalized to those of *Gapdh* and *Hprt* and analyzed via the 2^-ΔΔCT^ method.

### mRNA stability and translation inhibition

For mRNA stability assays, ILC2s were treated with 10 µM actinomycin D (Sigma, SBR00013) to inhibit transcription, and mRNA decay was monitored by qPCR. Half-lives were calculated via a nonlinear one-phase decay model. For the translation inhibition assays, 10 µM cycloheximide (Sigma, C4859-1 ml) was used, and the cytokine levels were measured via ELISA, while the transcription levels were assessed via qPCR via the 2-^ΔΔCT^ method.

### Puromycin incorporation assays

For puromycin incorporation, stimulated-ILC2s and lung single cells were treated with 10 µg/ml puromycin (InvivoGen, ant-pr-1) for 30 minutes before harvest. After surface staining, the cells were fixed and permeabilized via a Cytofix/Cytoperm Kit. Puromycin incorporation was detected by flow cytometry using AF647-conjugated anti-puromycin antibodies (BioLegend, 381508), and the cells were stained for IL-5 and IL-13 to assess translation efficiency.

### Immunofluorescence imaging of human and mouse ILC2s

For fluorescence imaging of human and mouse ILC2s, the cells were fixed with 2% paraformaldehyde and permeabilized with the Foxp3/Transcription Factor Staining Buffer Set. Mouse ILC2s were stained with AF488-conjugated anti-CD90.2 (BioLegend, 105311), anti-Piezo1 (Novus, NBP1-78446), APC-conjugated anti-IL-5 (BioLegend, 504306), PE-conjugated anti-IL-13 (Invitrogen, 12-7133-82), and ER-Tracker Green (Invitrogen, E34250) antibodies. Fixed human ILC2s were stained with anti-Piezo1 (Novus, NBP1-78446), AF647-conjugated anti-CD161 (BioLegend, 339910) and PE-conjugated anti-ST2 (BioLegend, 379404). ILC2 nuclei were stained with DAPI-containing medium (Invitrogen, P36961) or 1 ng/ml Hoechst (Invitrogen, H3750) in PBS for 15 min and mounted with Vectashield Antifade Mounting Medium (Vector Lab, H-1000-10). ILC2s were imaged via a Nikon A1 confocal microscope and an Andor BC43 benchtop confocal microscope (Oxford Instruments, UK). Image analysis was performed via Nikon NIS-Elements Viewer (Nikon, Japan) and Oxford Fusion and Imaris (Oxford Instruments, UK).

### Bulk mRNA sequencing

Purified murine lung ILC2s stimulated with Yoda1 for 3 hours were lysed, and RNA was extracted via TRIzol reagent. The RNA quality was assessed with a TapeStation 4000 (Agilent Technologies) and quantified via an ND-2000 Spectrophotometer (Thermo Fisher). mRNA was isolated with the Poly(A) RNA Selection Kit (LEXOGEN), and libraries were prepared via the NEBNext Ultra II Directional RNA-Seq Kit. The cDNA was indexed with Illumina indices and amplified via PCR. Libraries were checked for fragment size with TapeStation HS D1000 Screen Tape and quantified by real-time PCR (Life Technologies). Sequencing was performed on a NovaSeq 6000 (Illumina). Data quality control was performed with FastQC, and the reads were processed via FASTX Trimmer and BB Map. Gene expression was analyzed with Cufflinks, and DEGs were identified via Cuffdiff and GSEA 4.3.2. All the data were deposited in GEO (GSE278085).

### Single-cell ATAC sequencing and analysis

Total murine lung ILCs (Lin^-^CD127^+^) from tamoxifen-inducible Piezo1^fl/fl^ and Piezo1^fl/fl^ Id2-CreER^T2^ mice treated with rmIL-33 were sorted for single-cell ATAC and RNA sequencing via 10x Genomics platforms. For scATAC-seq, nuclei were prepared via the FL™ Automated Fluorescence Cell Counter (Logos Biosystems), transposed following the 10x Chromium Multiome ATAC+ Gene Expression protocol (CG000338), and sequenced on an Illumina HiSeq platform. Low-quality cells were filtered (nFrags > 1000 and TSS enrichment > 10). The doublets were removed via the filterDoublet function (filterRatio = 1.0) from ArchR (v1.0.2). The data were processed and normalized via the Cell Ranger ArchR pipeline. To reduce dimensionality (resolution = 0.2) and correct batch effects, we utilized the iterative LSI method and Harmony (v0.1.1). For UMAP visualization, we conducted Harmony-batch corrected reduced dimensions and cosine metrics. Gene scores were calculated via MACIC (v3.0.0). The ATAC signals normalized by ReadslnTSS were used to visualize the genome browser track results.

### Single-cell RNA sequencing and analysis

For scRNA-seq, single-cell suspensions were processed via the Next GEM 3’ v3.1 protocol (CG000315), sequenced on an Illumina HiSeq, and aligned via Cell Ranger (v6.0.1, 10x Genomics). Filtered gene‒cell matrices were analyzed with Seurat (v5.0.0) by applying quality filters (mitochondrial gene percentage <10%, detected genes >200 and <5000). Doublets were removed via DoubletFinder (v2.0.3). The data were normalized and variance-stabilized via SCTransform. Principal components were computed from the top 3000 highly variable genes and corrected for batch effects via Harmony (v0.1.1). UMAP visualization was performed via Harmony-corrected PCs with cosine distance, and clustering was performed via the Louvain algorithm (resolution = 0.3). Marker genes and differentially expressed genes (DEGs) were identified via the FindAllMarkers and FindMarkers functions (MAST, v1.25.2). Gene set enrichment analysis (GSEA) was performed via clusterProfiler (v4.6.2). Module scores were calculated with Seurat AddModuleScore. Pathways were obtained from the REACTOME and Gene Ontology (GO) databases. All the data were deposited in GEO (GSE279338).

### Public single-cell RNA sequencing data analysis

The mouse idiopathic pulmonary fibrosis (IPF) model dataset (GSE164220) was analyzed. ILCs extracted from the original dataset. Smart local moving (SLM) algorithm-based clustering was performed on defined ILC2 populations on the basis of canonical ILC2 marker gene expression. The ILC2 population from murine IPF data was divided into two groups, *Piezo1*-high/low, via K-means clustering (*k* = 2). For fetal lung atlas data (E-MTAB-11528), clustering and GSEA were performed similarly. Clustering and GSEA were done using standard Seurat and clusterProfiler tools, dividing ILC2 cells into *PIEZO1*-high/low groups via K-means (k = 2) clustering. Module scores were calculated via AddModuleScore, with pathways retrieved from the GO database.

### Murine models of lung inflammation and fibrosis

For the mouse lung inflammation model, recombinant mouse (rm) IL-33 (BioLegend, 580508) at 12.5 μg/kg was suspended in sterile PBS and administered intratracheally to lightly isoflurane-anesthetized mice. rmIL-33 was administered three times, and the mice were sacrificed on day 7 after the initial injection. *Alternaria. Alternata* (Greer, M1) at 250 μg/kg was suspended in sterile PBS and administered intratracheally to lightly isoflurane-anesthetized mice. A. A was administered three times, with the mice sacrificed on day 5 after the initial injection. For the mouse lung fibrosis model, bleomycin (BLM) (Nippon Kayaku Co., BLMI-3J006) at 3 U/kg was suspended in sterile PBS and administered intratracheally to lightly isoflurane-anesthetized mice. The mice were sacrificed on day 14 after the initial injection. For the mTOR inhibition in vivo murine model, 0.2 mg/kg rapamycin (Sigma, 553210) was intraperitoneally injected every 2 days during the experiments.

### Mouse lung histology and imaging

The lung lobes were fixed in 4% paraformaldehyde (Biosesang, P2031) and embedded in paraffin. Sections (4 µm) were stained with H&E, PAS, and Masson’s trichrome for histological analysis. Fibrosis was assessed by deparaffinizing and permeabilizing sections with 0.2% Triton X-100 (Sigma, T8787), followed by antigen retrieval. The sections were stained with primary antibodies and imaged via Nikon A1 confocal microscopy (Nikon, Japan) and an Andor BC43 benchtop confocal microscope (Oxford Instruments, UK). Fibrotic areas were quantified via ImageJ software (NIH, USA).

### Statistical analysis

The data are presented as the means ± standard errors of the means (SEMs). Statistical analyses were performed via GraphPad Prism 9.5.1. Comparisons between two groups were made via the Mann‒Whitney U test, whereas multiple comparisons were assessed via one-way or two-way ANOVA, depending on the number of groups and variables. For human-derived ILC2s treated with DMSO or Yoda1, a paired t-test was used. A nonlinear one-phase decay model was used for decay analysis. A *P* value of less than 0.05 was considered statistically significant.

## Supplementary information

Supplementary_Materials

Western blot original film

Supplementary Movie 1

Supplementary Movie 2

Supplementary Movie 3

Supplementary Movie 4

Supplementary Movie 5

Supplementary Movie 6

## Data Availability

Bulk mRNA sequencing data have been deposited in GSE278085 and are publicly available. Single-cell RNA and ATAC sequencing data have been deposited in GSE279338 and are publicly available. Any additional information required to reanalyze the data reported in this paper is available from the corresponding author upon request.
