# Supplementary material for: Piezo1-mediated mechanotransduction regulates the translational activity, function and lung pathogenicity of group 2 innate lymphoid cells
Source: Signal Transduct Target Ther. 2025 Aug 21;10:269. doi: 10.1038/s41392-025-02350-4 (PMC12370950; doi:10.1038/s41392-025-02350-4)
Supplement: Supplementary file 1 — Supplementary_Materials [file 41392_2025_2350_MOESM1_ESM.docx]

Supplementary Materials for

Piezo1-mediated mechanotransduction regulates translational activity, functions, and lung pathogenicity of group 2 innate lymphoid cells

MinYeong Lim^1,2^, Seonjun Park^3^, Yoon ha Joo^4^, Sung Eun Kim^5,6^, Min Hee Ham^7,8^, TaeSoo Kim^2^, Kihyuck Kwak^7,8,9^, Sung Joon Kim^5,10^, Jung Chan Lee^11,12^, Sung Ho Park^3^, Hye Young Kim^1,2,13,14*^

Correspondence to: [hykim11@snu.ac.kr](mailto:hykim11@snu.ac.kr) (H.Y.K.)

**This PDF file includes:**

Supplementary Materials and Methods

Figures. S1 to S17

Captions for Movies S1 to S6

**Other Supplementary Materials for this manuscript include the following:**

Movies S1 to S6

Original and uncropped films of Western blotting

Supplementary Materials and Methods.

Mouse single cells preparation

Whole tibiae, femora, and spleens were collected from euthanized mice. Bone marrow was flushed from the tibiae and femora using a syringe. The spleen was cleaned of surrounding tissue and gently crushed. The resulting cell suspensions from all tissues were filtered through a 40 µm strainer, treated with RBC lysis buffer (BioLegend, 420301), and resuspended in buffer for analysis.

Piezo1 stimulation using EGAT and EDTA

For Piezo1 modulation, ILC2s were treated with 5 µM Yoda1 (Tocris, 5586) and 2 mM calcium chelators (EGTA, Biosesang, ER2024-001-80; EDTA, Sigma, E7889) for designated time points.

Flowcytometric cell proliferation and apoptosis analysis

For cell division and proliferation assays, purified ILC2s were labeled with 10 µM CFSE (Thermo Fisher Scientific, C34554) and cultured in the presence of rmIL-2, -7, and -33 (10 ng/mL each). After 72 hours, CFSE^+^ and Ki67^+^ ILC2s were analyzed. Apoptotic cells were identified by staining Annexin V with staining buffer (BioLegend, 422201). Flow cytometry data were analyzed using FlowJo software v10 (BD, NJ, USA) with BD LSR Fortessa™ X-20 and BD LSRII™ (BD, NJ, USA) instruments.

Whole-Cell Patch Clamp for SK4 channel

Whole-cell patch-clamp recordings were performed using an Axopatch-200B amplifier and pCLAMP software. The pipette solution for SK4 channel recording contained 140 mM KCl, 5 mM NaCl, 10 mM HEPES, 5 mM EGTA, 0.5 mM MgCl_2_, and 3 mM MgATP (pH 7.2) with a free Ca^2+^ concentration of 500 nM. The bath solution contained 145 mM NaCl, 3.6 mM KCl, 10 mM HEPES, 5 mM glucose, 1 mM MgCl_2_, and 1.3 mM CaCl_2_ (pH 7.4).

*A. Alternata*-induced muurine asthma models

*Alternaria. Alternata* (*A.A*) (Greer, M1) at 250 μg/kg was suspended in sterile PBS and administered intratracheally to lightly isoflurane-anesthetized mice. *A.A* was administered three times, with mice sacrificed on day 5 after the initial injection.

Figure. S1.


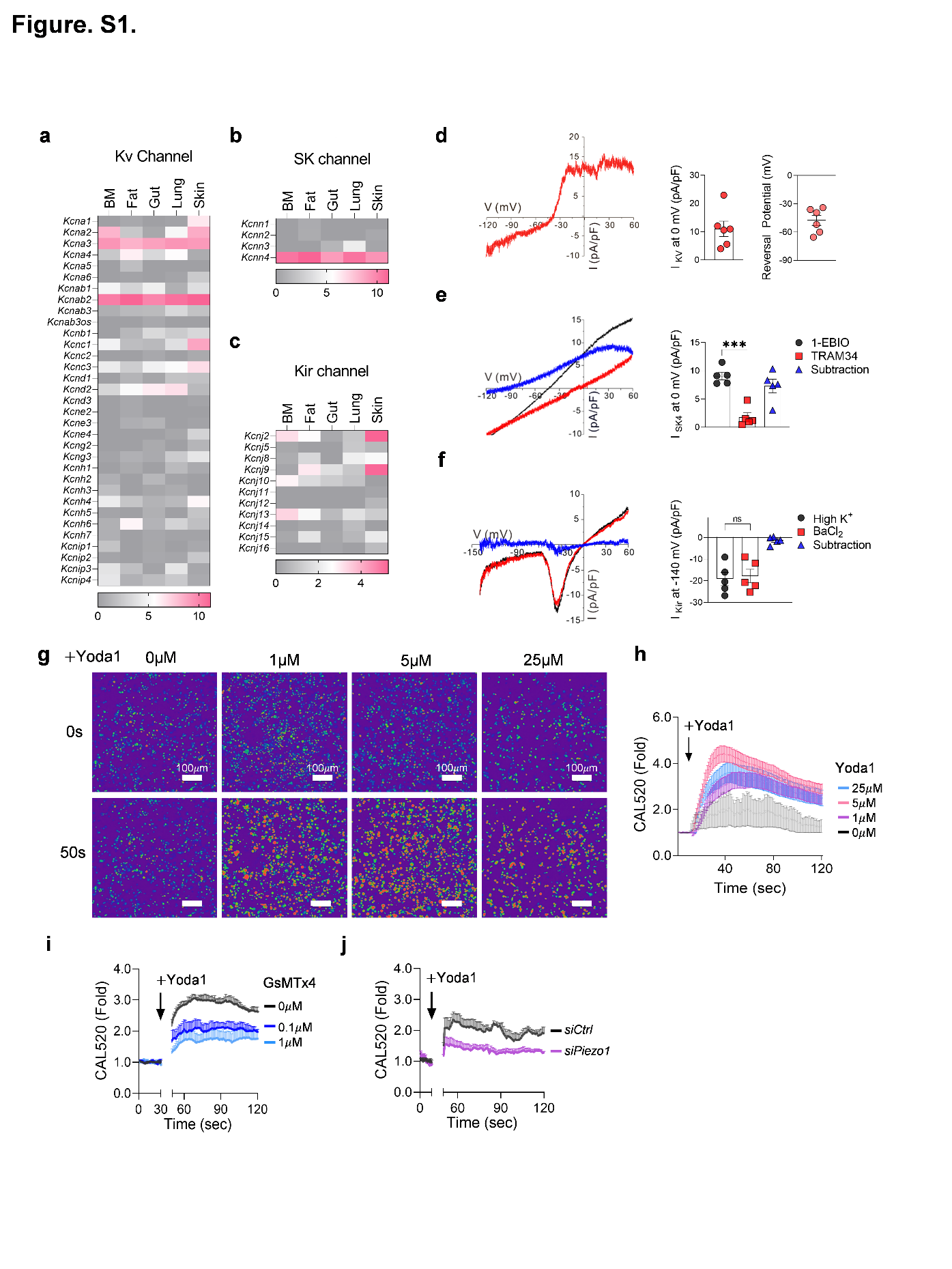


**Supplementary Figure 1 | Expression of potassium channels and validation of Piezo1-mediated Ca²⁺ influx in lung ILC2s.**

(a–c) Heatmaps showing the expression of Kv (a), SK (b), and Kir (c) potassium channels in tissue-resident ILC2s from bone marrow (BM), fat, gut, lung, and skin (Roberto et al., 2018). (d–f) Current–voltage (I/V) relationships and summary of current densities in murine lung ILC2s: (d) IKv measured at 0 mV (n=6), with reversal potential –47.85 ± 5.4 mV; (e) ISK4 measured at 0 mV with 1-EBIO and TRAM34 (n=5); (f) IKir measured at –140 mV under high K⁺ and BaCl₂ conditions (n=5). (g) Representative CAL-520 AM–labeled live-cell images of ILC2s at baseline and 50 seconds after Yoda1 stimulation (0–25 μM) (Scale bars = 100 µm). (h) Quantification of intracellular Ca²⁺ levels (CAL-520 AM fold change) in response to Yoda1 (n=32). (i) Flow cytometry analysis showing that Yoda1-induced Ca²⁺ influx is blocked by GsMTx4 (n=2). (j) Flow cytometry analysis showing that Yoda1-induced Ca²⁺ influx is abolished in *siPiezo1*-transfected ILC2s compared to *siCtrl* (n=3). Statistical significance was determined using One-way ANOVA as appropriate. Data are presented as mean ± SEM and are pooled from at least two to three independent experiments. ***P < 0.001; ns, not significant. Type or paste caption here. Create a page break and paste in the Figure above the caption.

Figure. S2.


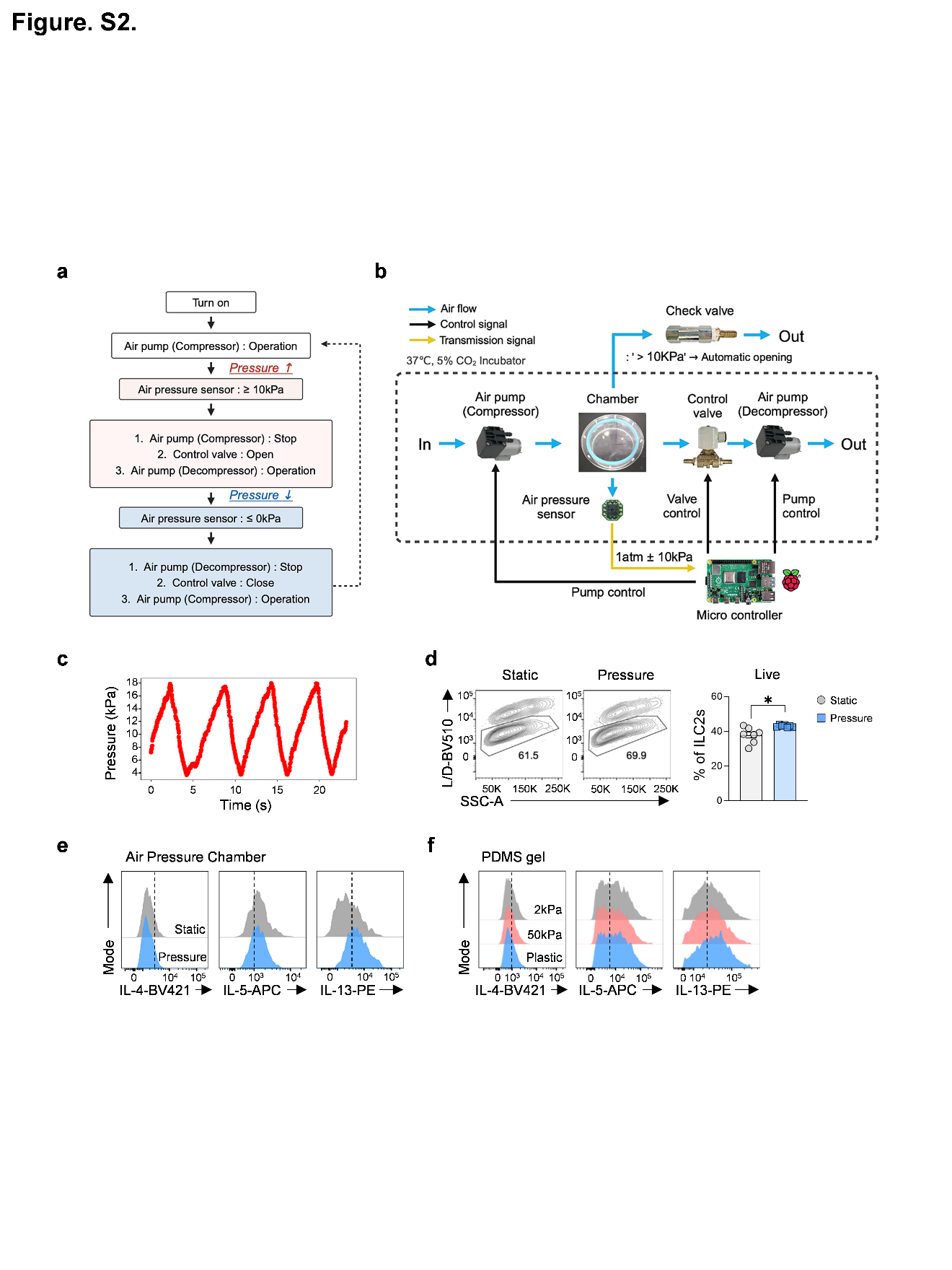


**Supplementary Figure 2 | Mechanical stimulation *via* cyclic air pressure or substrate stiffness enhances IL-13 production in ILC2s.**

(a, b) Schematic diagrams of the custom-built cyclic air pressure (CAP) system mimicking murine respiratory motion. (a) Programmable cyclic air compression and decompression generating ± 10 kPa oscillations. (b) System components including compressor, decompressor, air pressure sensor, control valves, and incubator. (c) Representative waveform illustrating cyclic pressure fluctuations over time. (d) Representative flow cytometry plots and quantification of viable ILC2s cultured under static or cyclic pressure conditions (n=7). (e) Representative histograms of intracellular IL-4, IL-5, and IL-13 expression in ILC2s under static or cyclic pressure conditions. (f) Representative histograms showing IL-4, IL-5, and IL-13 expression in ILC2s cultured on substrates of varying stiffness: 2 kPa PDMS, 50 kPa PDMS, and plastic ( > 1 GPa). Statistical significance was determined using the Mann–Whitney U-test. Data are pooled from at least two to three independent experiments and are presented as mean ± SEM. *P < 0.05.

**Figure. S3.**


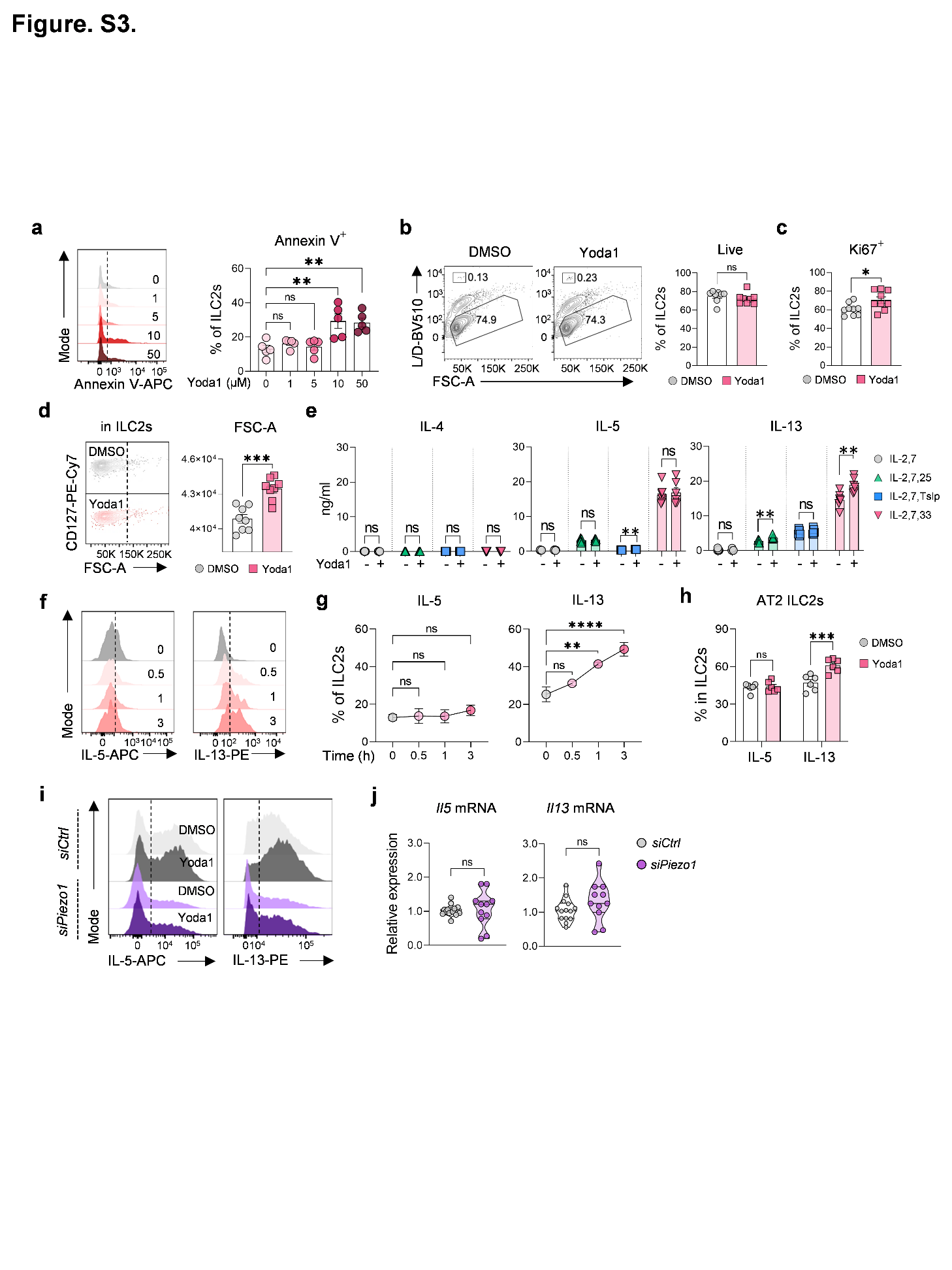


**Supplementary Figure 3 | Piezo1 activation promotes ILC2 functionality and enhances IL-13 protein production.**

(a) Representative histograms and quantification of Annexin V⁺ apoptotic ILC2s after 3-hour stimulation with increasing concentrations of Yoda1 (0–50 μM) (n=5). (b) Representative flow cytometry plots and quantification of live ILC2s (LD⁻ FSC-A⁺) following 72-hour stimulation with 5 μM Yoda1 (n=8). (c) Frequencies of Ki67⁺ ILC2s after 3-hour Yoda1 treatment (n=9). (d) Forward scatter area (FSC-A) as a proxy for cell size in DMSO- and Yoda1-treated ILC2s (n=8).

(e) ELISA of IL-4, IL-5, and IL-13 in culture supernatants of ILC2s stimulated with Yoda1 (5 μM) in combination with IL-2, IL-7, IL-25, IL-33, or TSLP (n=6–7). (f, g) Time-course analysis of IL-5⁺ and IL-13⁺ ILC2s following Yoda1 stimulation, measured by intracellular cytokine staining (n=6). (h) Frequencies of IL-5⁺ and IL-13⁺ ILC2s from adipose tissue (AT2 ILC2s) (n=6). (i) Representative histograms showing IL-5 and IL-13 expression in ILC2s transfected with *siCtrl* or *siPiezo1* following Yoda1 treatment. (j) qPCR analysis of *Il5* and *Il13* mRNA levels in *siCtrl*- and *siPiezo1*-transfected ILC2s (n=11–14). Statistical significance was determined using the Mann–Whitney U-test, one-way ANOVA, or two-way ANOVA, as appropriate. Data are pooled from two to three independent experiments and presented as mean ± SEM. *P < 0.05, **P < 0.01, ***P < 0.001, ****P < 0.0001; ns, not significant.

Figure. S4.


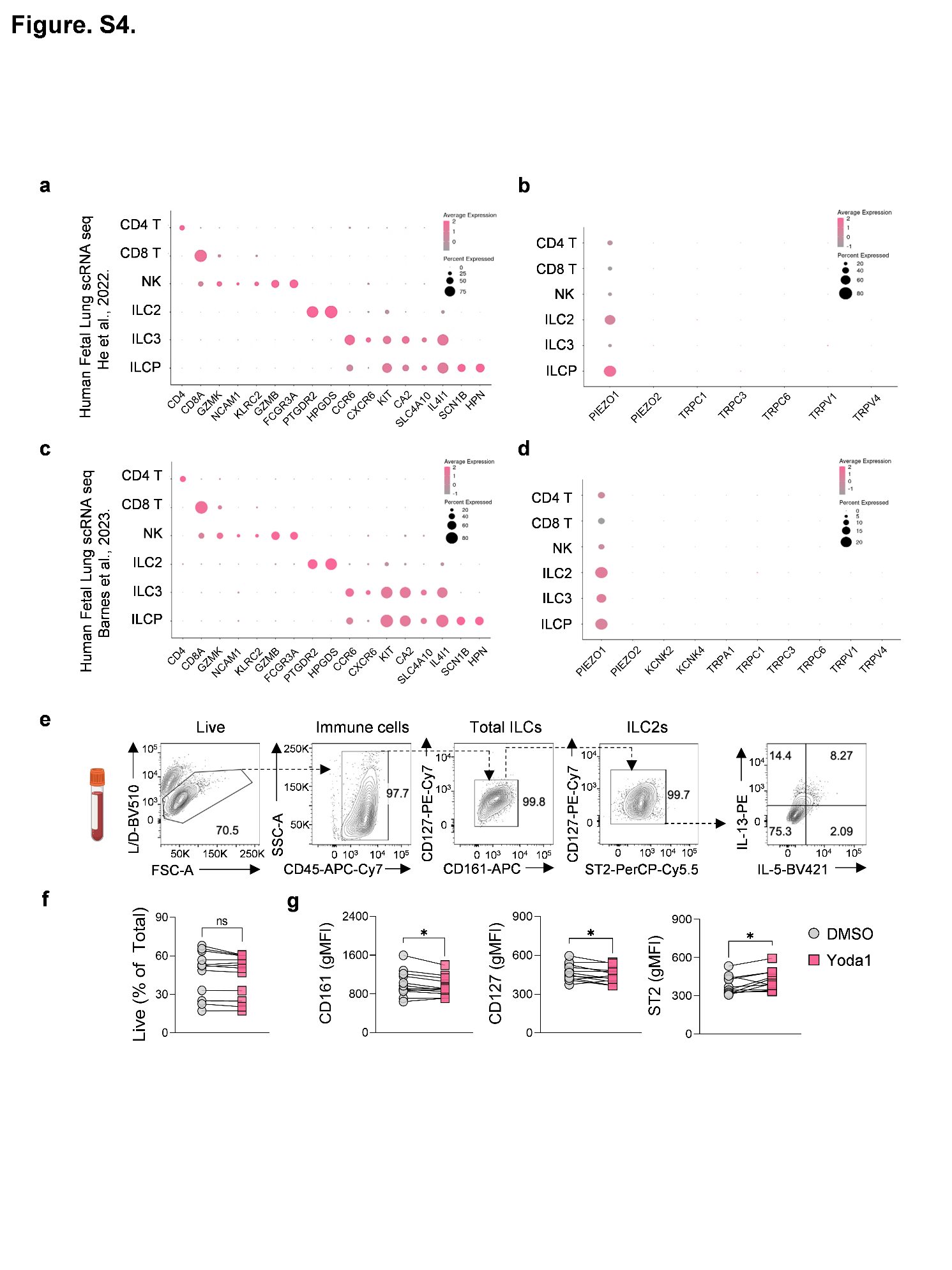


**Supplementary Figure 4 | *PIEZO1* is highly expressed in human ILC2s and regulates surface marker expression in human peripheral blood–derived ILC2s.**

(a–d) Public human fetal lung scRNA-seq analysis from He et al. (2022) (a, b) and Barnes et al. (2023) (c, d). (a, c) Dot plots showing average and percent expression of lymphocyte lineage markers in CD4⁺ T cells, CD8⁺ T cells, NK cells, ILC2s, ILC3s, and ILCPs. (b, d) Dot plots showing expression levels of mechanosensitive ion channels including *PIEZO1*, *PIEZO2*, *TRPCs*, and *TRPVs* across the same subsets. (e) Gating strategy for identifying human peripheral blood ILC2s and intracellular cytokine staining after 3-hour stimulation with Yoda1 (5 μM). (f) Frequencies of live ILC2s following DMSO or Yoda1 treatment (n=12). (g) Geometric mean fluorescence intensity (gMFI) of CD161, CD127, and ST2 in Yoda1-treated ILC2s (n=12). Statistical significance was determined using the paired t-test. Data are pooled from at least two to three independent experiments. *P < 0.05; ns, not significant.

Figure. S5.


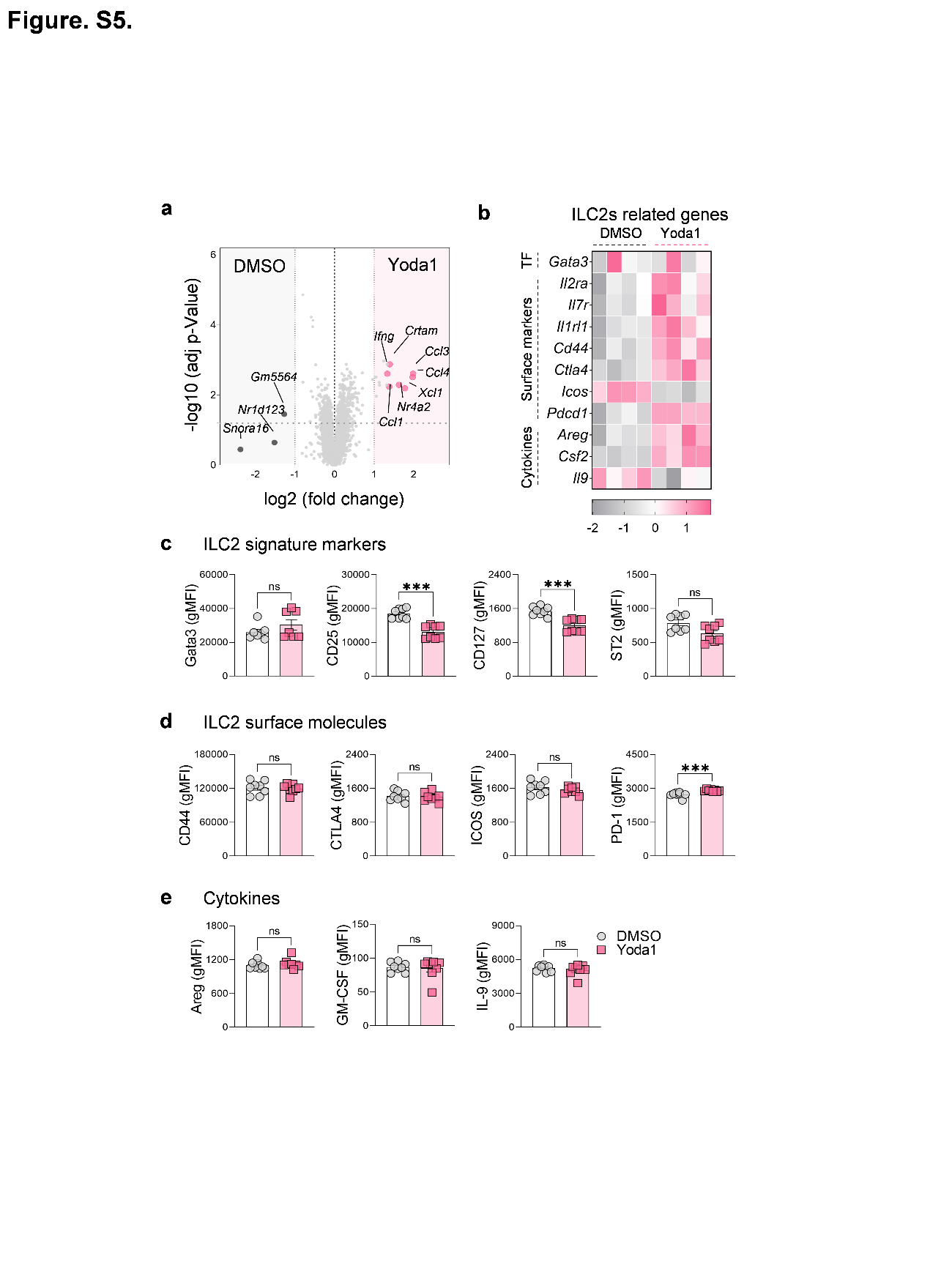


**Supplementary Figure 5 | Transcriptomic and phenotypic analysis of ILC2s upon Piezo1 activation.**

(a) Volcano plot of differentially expressed genes from bulk RNA-seq comparing DMSO- and Yoda1-treated murine lung ILC2s (5 μM, 3 h; n=4). Selected upregulated genes such as *Ccl3, Ccl4*, and *Xcl1* are highlighted. (b) Heatmap showing relative expression (Z-score) of transcription factors, surface markers, and cytokine-related genes in DMSO- vs. Yoda1-treated ILC2s. (c–e) Flow cytometric quantification of geometric mean fluorescence intensity (gMFI) for selected markers and cytokines in ILC2s (n=7–8): (c) GATA3, CD25, CD127, ST2; (d) CD44, CTLA-4, ICOS, PD-1; (e) Areg, GM-CSF, and IL-9. Statistical significance was determined using the Mann–Whitney U-test. Data are pooled from two to three independent experiments and presented as mean ± SEM. ***P < 0.001, ****P < 0.0001; ns, not significant.

Figure. S6.


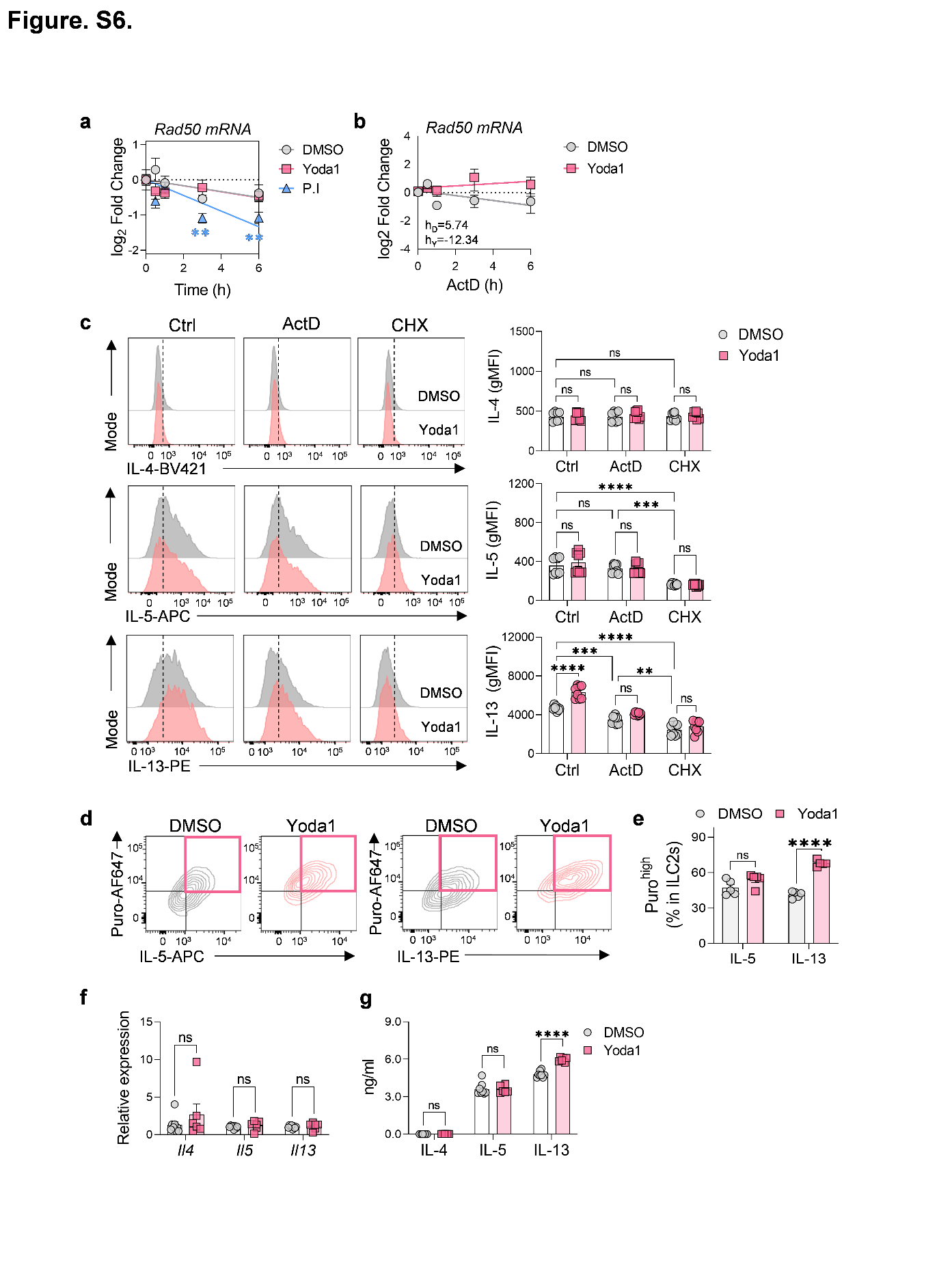


**Supplementary Figure 6 | Piezo1 activation selectively stabilizes *Il13* mRNA and enhances IL-13 protein translation in ILC2s.**

(a) Time-course of *Rad50* mRNA expression in ILC2s treated with DMSO, Yoda1 (5 μM), or PMA (100 ng/mL) + ionomycin (1 μg/mL), measured by qPCR (n=6–15). (b) *Rad50* mRNA decay kinetics following Actinomycin D (ActD, 1 μg/mL) treatment, showing increased transcript stability in Yoda1-treated ILC2s (n=11–12). (c) Representative histograms and gMFI of intracellular IL-4, IL-5, and IL-13 in ILC2s treated with DMSO or Yoda1, with or without ActD or cycloheximide (CHX), indicating post-transcriptional regulation of IL-13 (n=8). (d) Representative flow cytometry plots showing puromycin incorporation and co-expression of IL-5 or IL-13 in translating ILC2s (n=5). (e) Quantification of IL-5⁺ and IL-13⁺ Puro ^high^ ILC2s (n=5). (f) Relative mRNA expression of *Il4, Il5*, and *Il13* in DMSO- vs. Yoda1-treated ILC2s (n=6–8). (g) Cytokine concentrations of IL-4, IL-5, and IL-13 in culture supernatants from DMSO- or Yoda1-treated ILC2s measured by ELISA (n=6–8). Statistical significance was determined using two-way ANOVA and one phase decay for calculating mRNAs half-life .Data are pooled from at least two to three independent experiments or representative data and presented as mean ± SEM. **P < 0.01, ***P < 0.001, ****P < 0.0001; ns, not significant.

Figure. S7.


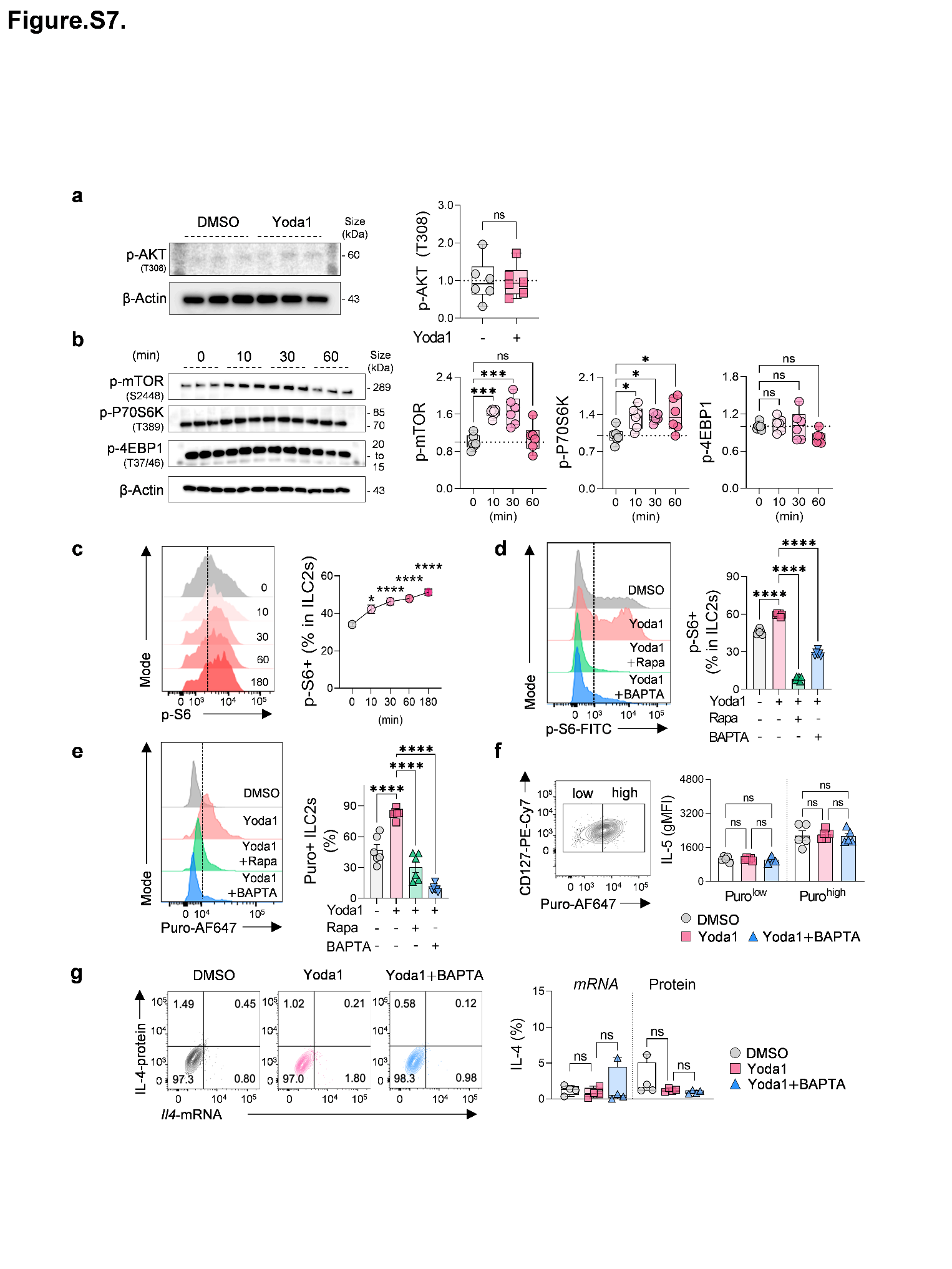


**Supplementary Figure 7 | Piezo1-mediated translation enhancement in ILC2s is dependent on Ca²⁺ influx and mTOR–S6 signaling.**

(a) Western blot and quantification of phosphorylated AKT (Thr308) in ILC2s treated with DMSO or Yoda1 (5 μM, 3 h) (n=6). (b) Time-course western blot analysis and quantification of phosphorylation of mTOR (Ser2448), p70S6K (Thr389), and 4E-BP1 (Thr37/46) following Yoda1 stimulation (n=6). (c) Representative histograms and time-course analysis of phosphorylated S6 (p-S6) in ILC2s treated with Yoda1, measured by flow cytometry (n=7–13). (d) Frequencies of p-S6⁺ ILC2s under DMSO, Yoda1, Yoda1 + rapamycin (100 nM), and Yoda1 + BAPTA (2 mM) conditions, assessed by flow cytometry (n=6). (e) Representative histograms and quantification of puromycin⁺ ILC2s under the same treatment conditions (n=6). (f) Representative flow cytometry plots and gMFI of IL-5 expression in Puro^low^ vs. Puro^high^ ILC2s under DMSO, Yoda1, or Yoda1 + BAPTA (n=5). This plot shows the gating strategy for the representative data displayed in Figure 3h. (g) Representative PrimeFlow™ cytometry plots showing *Il4* mRNA and IL-4 protein expression in ILC2s treated with DMSO, Yoda1, or Yoda1 + BAPTA (n=4). Statistical significance was determined using Mann–Whitney U-test, one-way ANOVA, or two-way ANOVA, as appropriate. Data are pooled from at least two to three independent experiments or representative experiments and presented as mean ± SEM. *P < 0.05, ***P < 0.001, ****P < 0.0001; ns, not significant.


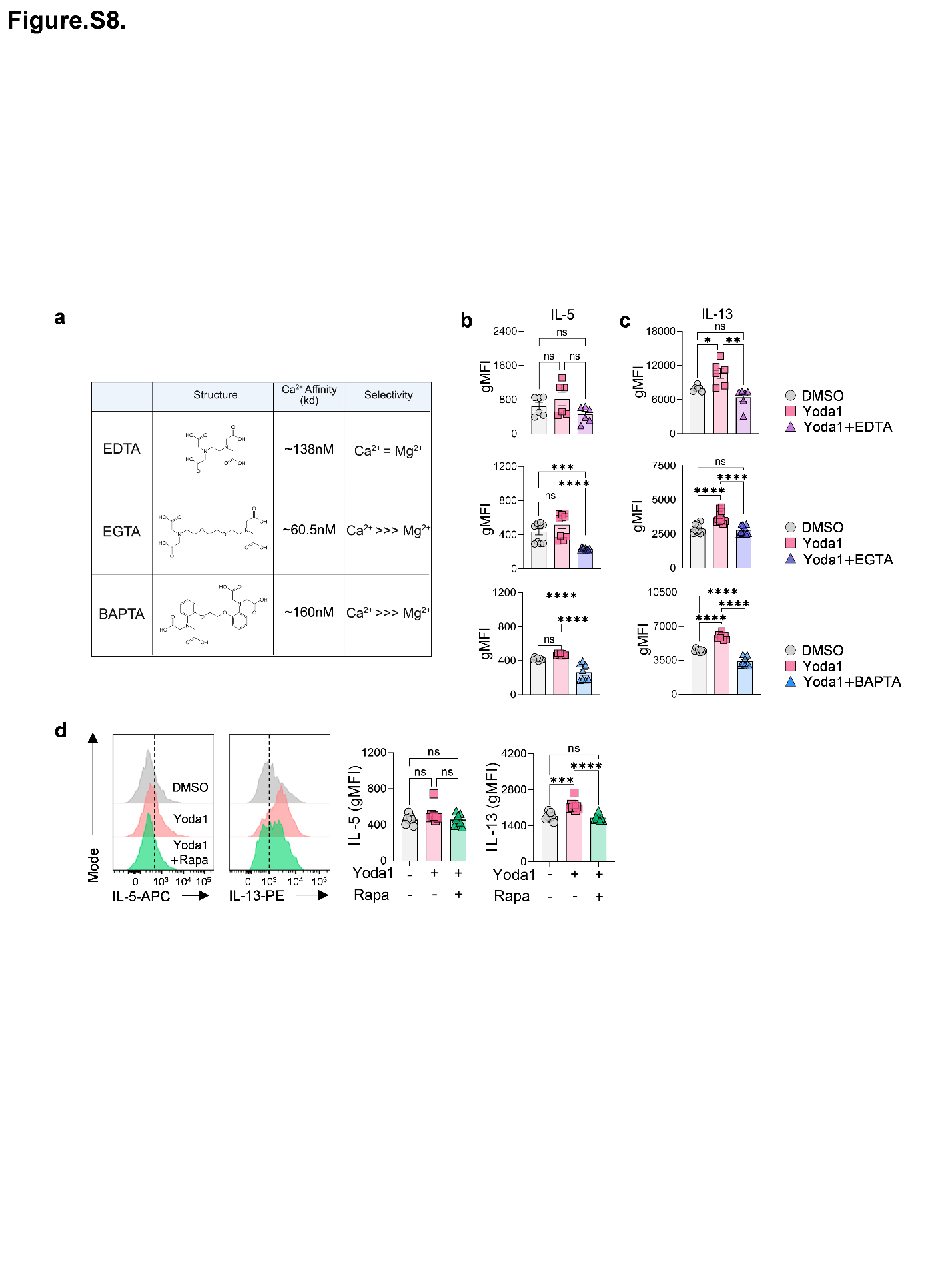
Figure. S8.

**Supplementary Figure 8 | Ca²⁺ influx and mTOR signaling are required for Piezo1-mediated IL-13 production in ILC2s.**

(a) Comparison of calcium chelators—EDTA, EGTA, and BAPTA—highlighting their chemical structures, calcium binding affinities (Kd), and Ca²⁺ vs. Mg²⁺ selectivity. (b, c) Geometric mean fluorescence intensity (gMFI) of intracellular IL-5 (b) and IL-13 (c) in ILC2s treated with DMSO, Yoda1 (5 μM), or Yoda1 combined with calcium chelators (EDTA, EGTA, or BAPTA; 2 mM each) for 3 hours (n=6–10). (d) Representative histograms and gMFI of IL-5 and IL-13 in ILC2s treated with DMSO, Yoda1, or Yoda1 + rapamycin (100 nM) (n=7–8). Statistical significance was determined using one-way ANOVA. Data are pooled from at least two to three independent experiments and presented as mean ± SEM. *P < 0.05, **P < 0.01, ***P < 0.001, ****P < 0.0001; ns, not significant.


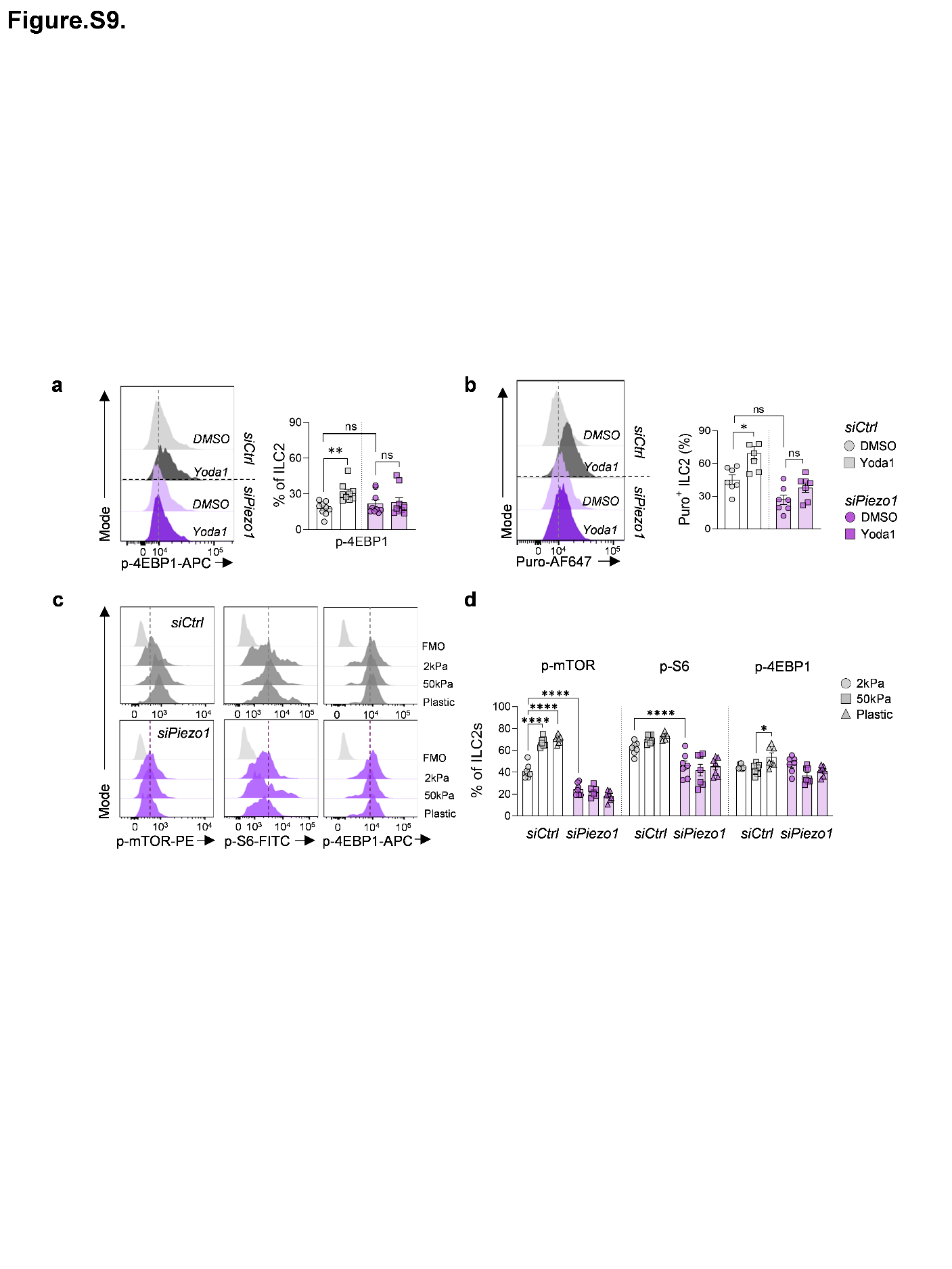
Figure. S9.

**Supplementary Figure 9 | Piezo1 is required for stiffness-dependent mTOR activation and translational activity in ILC2s**

(a) Representative histograms and quantification of p-4E-BP1 expression in control (*siCtrl*) or Piezo1-knockdown (*siPiezo1*) ILC2s treated with DMSO or Yoda1 (5 μM, 3 h) (n=9). (b) Representative histograms and quantification of puromycin⁺ ILC2s under the same conditions as in (a) (n=7). (c) Representative histograms and quantification of p-mTOR, p-S6, and p-4E-BP1 in control (*siCtrl*) or Piezo1-knockdown (*siPiezo1*) ILC2s cultured on PDMS substrates of different stiffness (2 kPa, 50 kPa, or plastic) (n=10). Statistical significance was determined using two-way ANOVA. Data are pooled from two to three independent experiments and presented as mean ± SEM. *P < 0.05, **P < 0.01, ****P < 0.0001; ns, not significant.

Figure. S10.

**
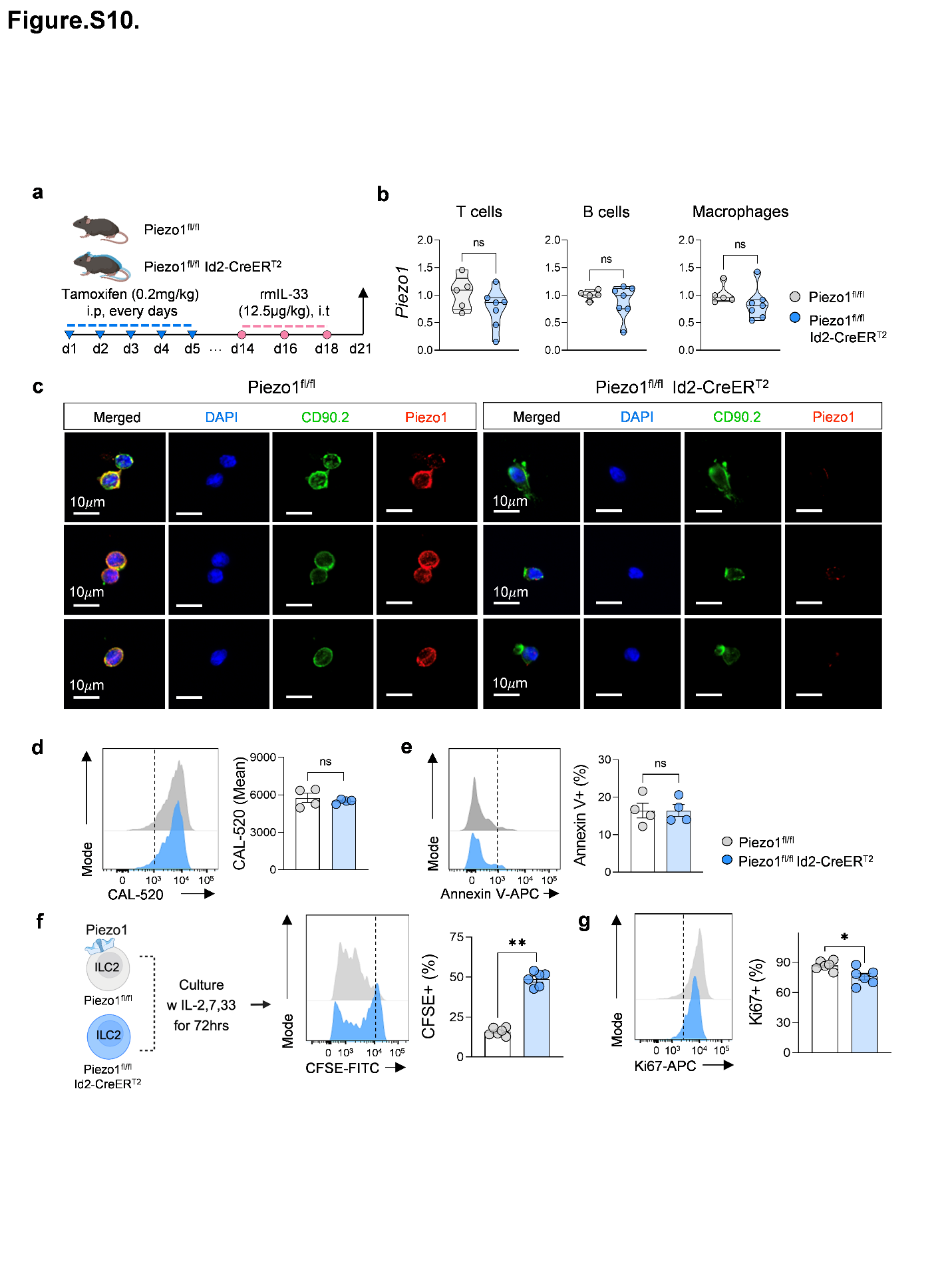
**

**Supplementary Fig. 10 | Validation of Piezo1 deletion and functional consequences in ILC2s from Piezo1^fl/fl^ and Piezo1^fl/fl^ Id2CreER^T2^ (cKO) mice.**

(a) Schematic of tamoxifen-induced Piezo1 deletion in ILC2s and the timeline for recombinant IL-33 administration. (b) qPCR analysis of *Piezo1* mRNA expression in sorted T cells, B cells, and macrophages from naïve Piezo1^fl/fl^ and cKO mice (n=5–7). (c) Representative confocal immunofluorescence images of CD90.2⁺ ILC2s stained for Piezo1 (red), CD90.2 (green), and DAPI (blue) from Piezo1^fl/fl^ and cKO mice. (Scale bars = 10 µm) (d) Representative histograms and quantification of intracellular Ca²⁺ response using CAL-520 AM in ILC2s from Piezo1^fl/fl^ and cKO mice (n=6). (e) Annexin V staining and quantification of apoptosis in ILC2s (n=6). (f, g) Cell proliferation assessed by CFSE dilution (f) and Ki67 expression (g) after 72-hour culture in IL-2/IL-7/IL-33 (n=6). Statistical significance was assessed using the Mann–Whitney U-test. Data are pooled from two to three independent or representative experiments and presented as mean ± SEM. *P < 0.05, **P < 0.01; ns, not significant.

Figure. S11.


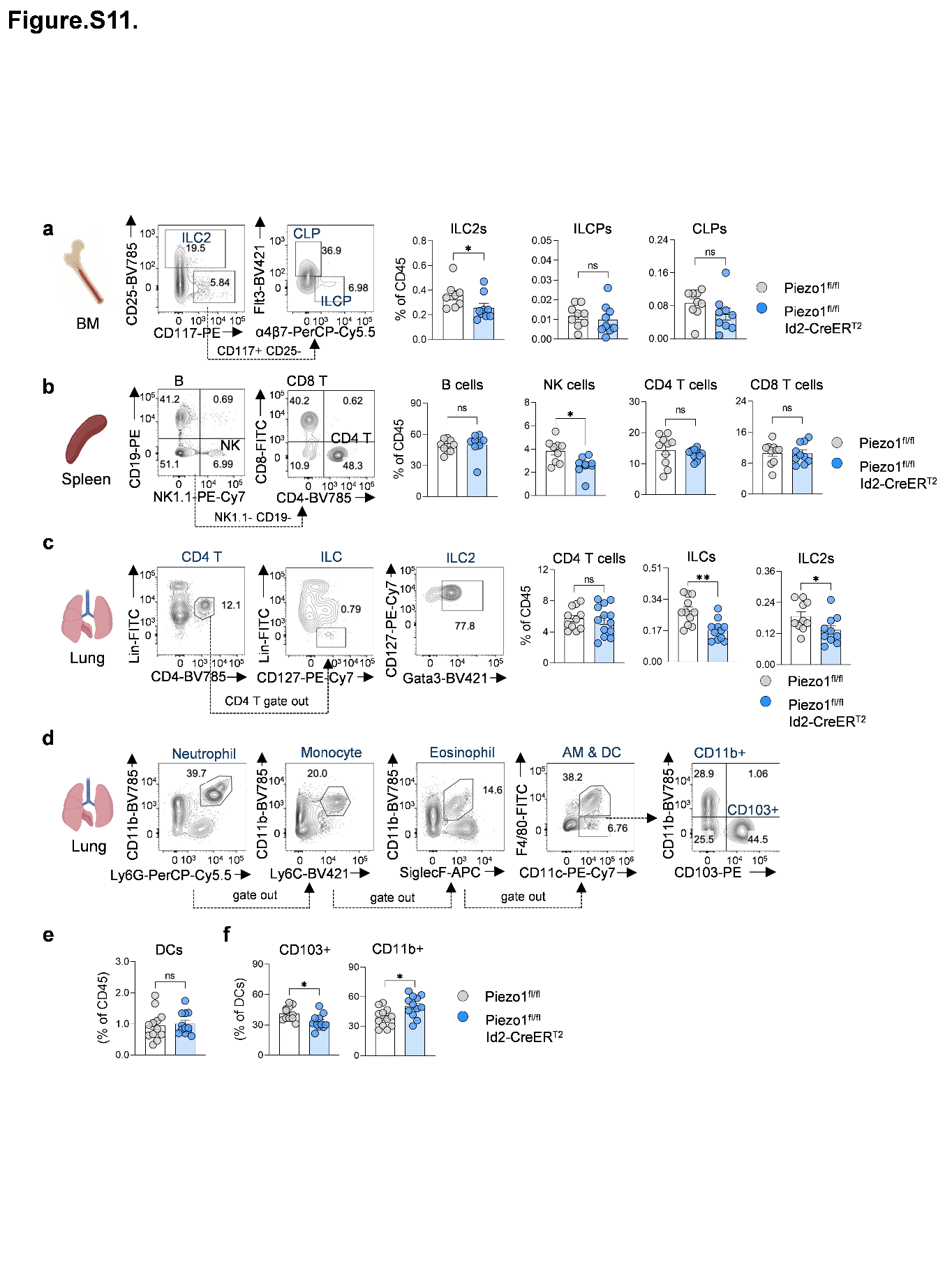

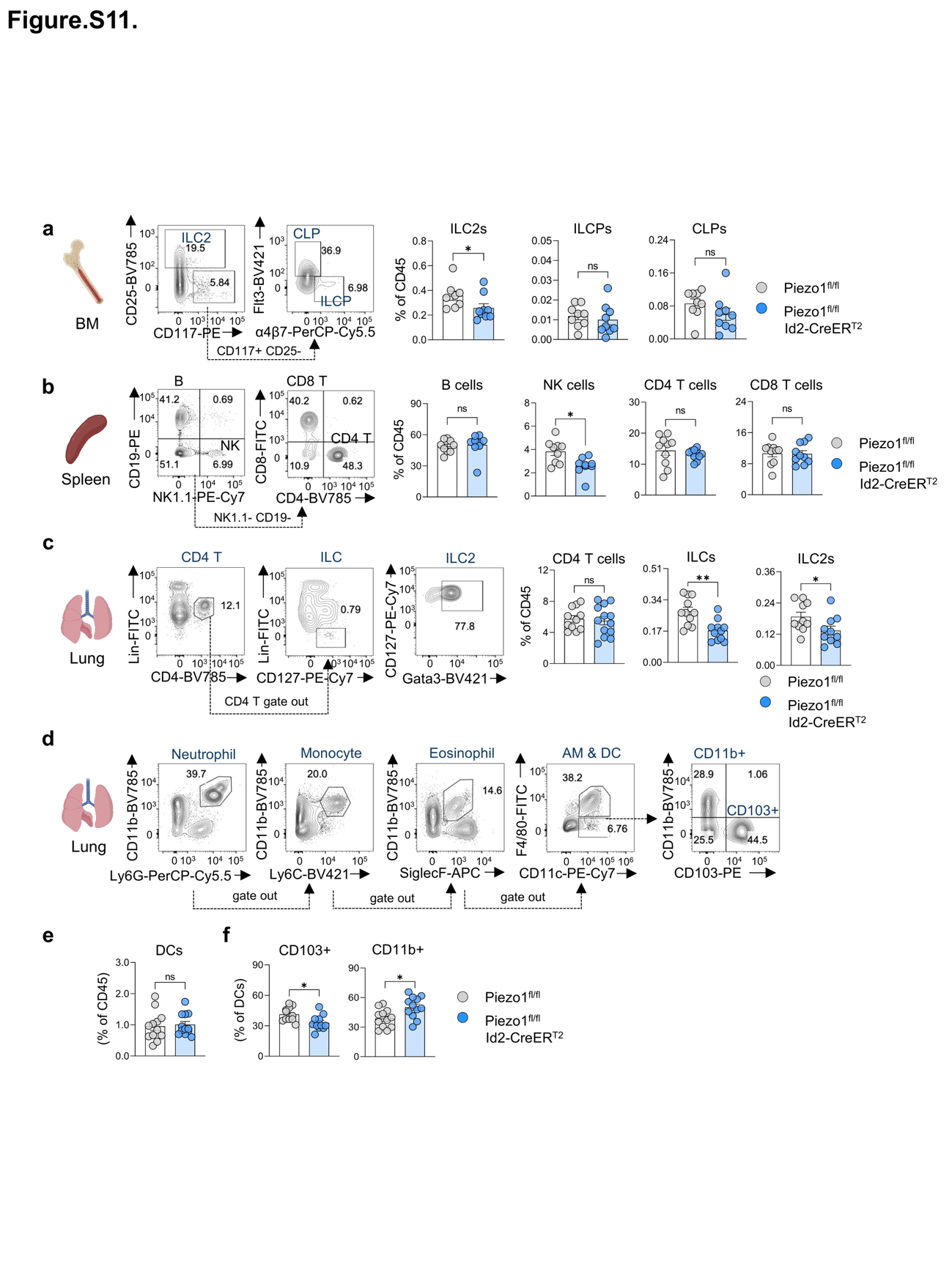


**Supplementary Fig. S11 | Immune cells profiling of bone marrow, spleen, and lung naïve Piezo1^fl/fl^ and Piezo1^fl/fl^ Id2CreER^T2^ (cKO) mice.**

(a) Representative flow cytometry plots and quantification of bone marrow ILC2s (Lin⁻ CD127⁺ CD25⁺ ST2⁺), ILCPs (α4β7⁺ Flt3⁻), and CLPs (α4β7^-^ Flt3⁺) in Piezo1^fl/fl^ and cKO mice (n=8–9). (b) Frequencies of splenic B cells (CD19⁺), NK cells (NK1.1⁺), and CD4⁺ and CD8⁺ T cells (n=9–10). (c) Gating strategy and quantification of lung immune subsets: ILCs (Lin⁻ CD127⁺), ILC2s (Lin⁻ CD127⁺ GATA3⁺), and CD4⁺ T cells (Lin⁻ CD4⁺) (n=10–13). (d) Representative flow cytometry plots showing gating strategy for lung neutrophils (Ly6G⁺), monocytes (Ly6C⁺), eosinophils (SiglecF⁺), alveolar macrophages (F4/80⁺ CD11c⁺), and dendritic cells (DCs: CD11c⁺ F4/80⁻), including CD103⁺ and CD11b⁺ subsets. (e, f) Quantification of total DCs among CD45⁺ lung cells (e), and the proportions of CD103⁺ and CD11b⁺ DC subsets (f) (n=11–12). Statistical significance was assessed using the Mann–Whitney U-test. Data are pooled from two to three independent experiments and presented as mean ± SEM. *P < 0.05, **P < 0.01; ns, not significant.


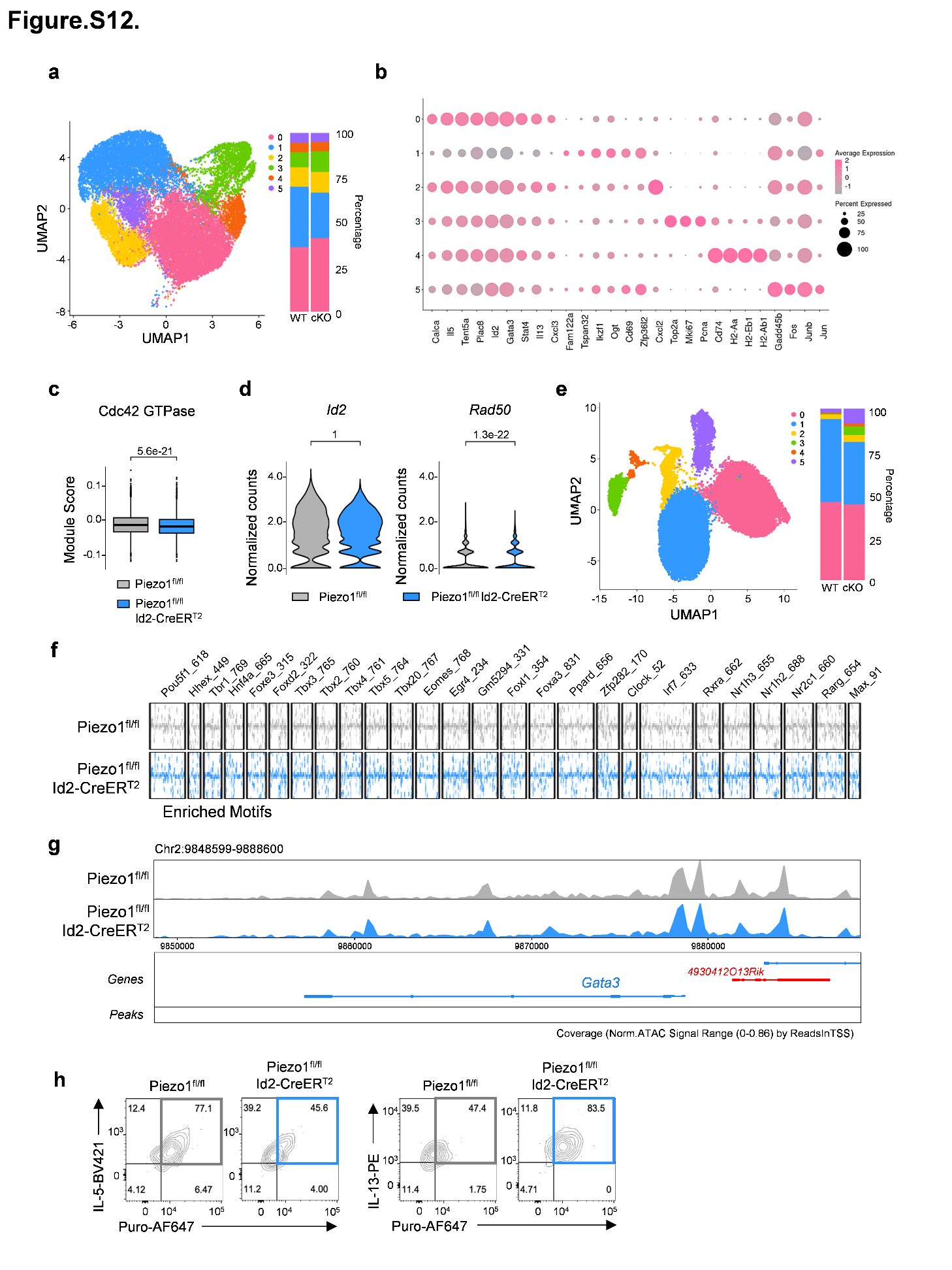
Figure. S12.

**Supplementary Fig. 12 | Integrated single-cell transcriptomic and chromatin accessibility analysis of lung ILC2s from Piezo1^fl/fl^ and Piezo1^fl/fl^ Id2CreER^T2^ (cKO) mice.**

(a) UMAP projection of scRNA-seq profiles showing distinct transcriptional clusters between Piezo1^fl/fl^ and cKO lung ILC2s. (b) Dot plots of selected marker gene expression across identified clusters. (c) Box plot comparing CDC42 GTPase module scores between Piezo1^fl/fl^ and cKO ILC2s. (d) Violin plots showing normalized expression of *Id2* and *Rad50* in Piezo1^fl/fl^ and cKO ILC2s. (e) UMAP projection of scATAC-seq profiles illustrating chromatin accessibility clustering in Piezo1^fl/fl^ and cKO ILC2s. (f) Motif enrichment analysis of transcription factor binding sites in accessible chromatin regions between genotypes. (g) Genome browser tracks showing chromatin accessibility at the *Gata3* locus, indicating reduced accessibility in Piezo1-deficient ILC2s. (h) Representative flow cytometry plots showing IL-5⁺ and IL-13⁺ populations among puromycin⁺ (translating) ILC2s in Piezo1^fl/fl^ and cKO mice.

Figure. S13.


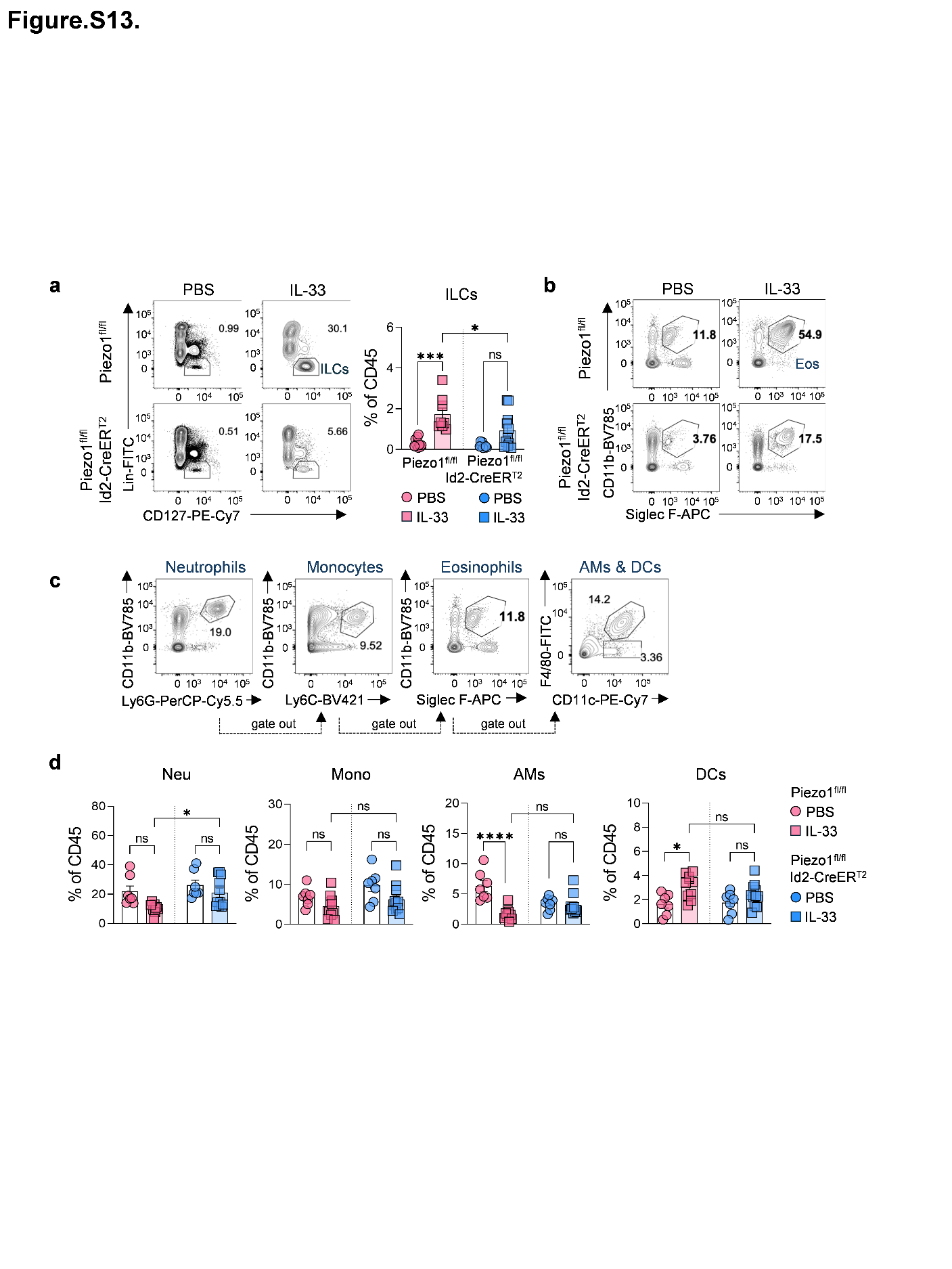


**Supplementary Fig. 13 | Immune cell profiling in Piezo1^fl/fl^ and Piezo1^fl/fl^ Id2CreER^T2^ (cKO) mice following IL-33-induced acute lung inflammation.**

(a) Representative flow cytometry plots and quantification of total lung ILCs (Lin⁻ CD127⁺) in Piezo1^fl/fl^ and cKO mice treated with PBS or IL-33 (n=8–10). (b) Representative plots showing eosinophil populations (CD11b⁺ SiglecF⁺) under the same conditions. (c) Gating strategy for lung neutrophils (Ly6G⁺ CD11b⁺), monocytes (Ly6C⁺ CD11b⁺), eosinophils (SiglecF⁺ CD11b⁺), alveolar macrophages (F4/80⁺ CD11c⁺), and dendritic cells (DCs; CD11c⁺ F4/80⁻). The FACS plots show the gating strategy for the representative data displayed in Figure S13b. (d) Quantification of neutrophils, monocytes, alveolar macrophages, and dendritic cells as a percentage of CD45⁺ lung cells (n=7–10). Statistical significance was determined using one-way ANOVA. Data are pooled from two to three independent experiments and presented as mean ± SEM. *P < 0.05, ****P < 0.0001; ns, not significant.

Figure. S14.


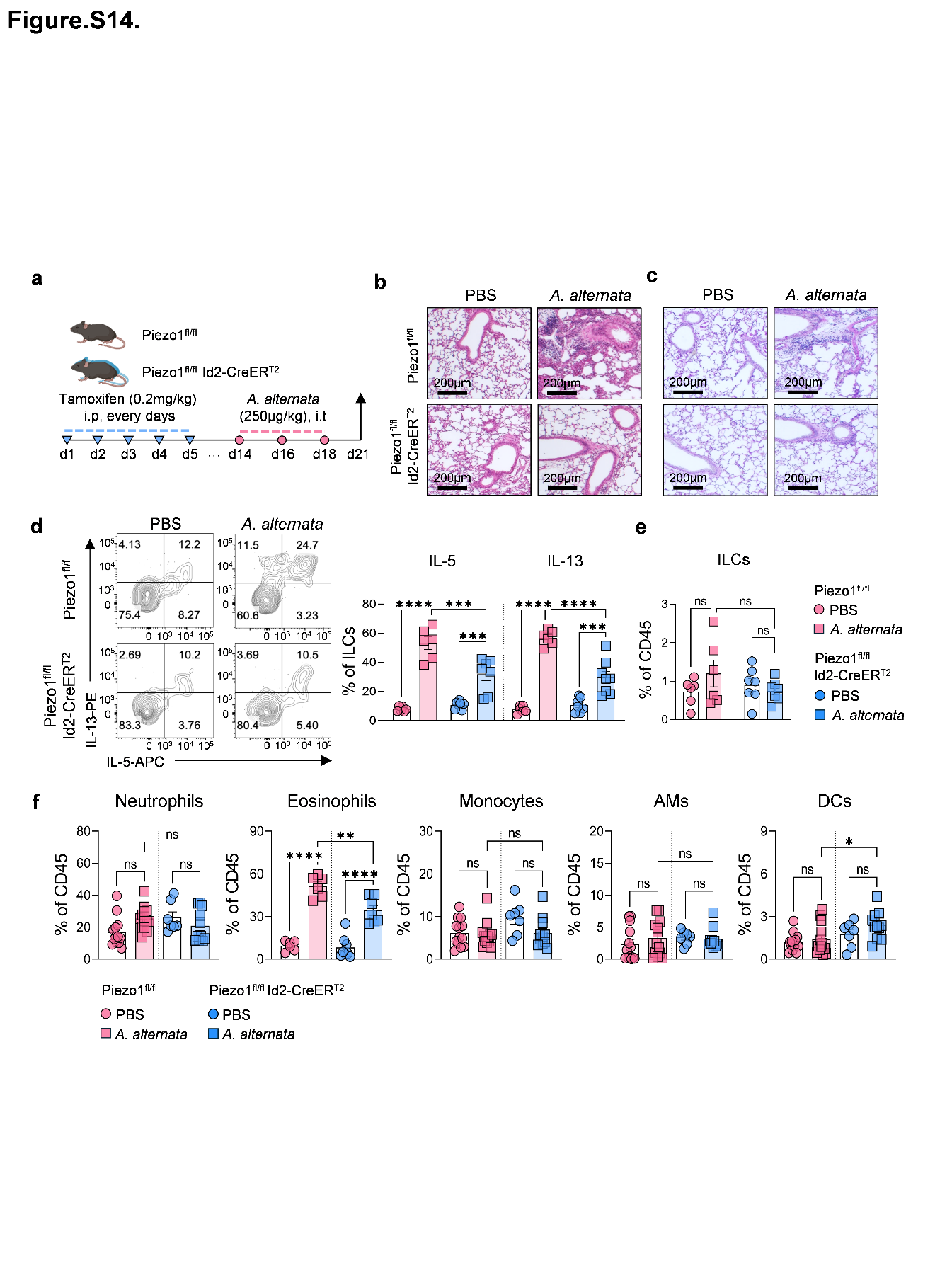


**Supplementary Figure 14 | Conditional deletion of Piezo1 in ILC2s attenuates type 2 inflammation in the *Alternaria alternata*–induced lung inflammation model.**

(a) Schematic of the experimental timeline: tamoxifen-induced Piezo1 deletion followed by intratracheal administration of A. alternata (250 μg/kg) every 2 days. (b, c) Representative lung histology showing H&E (b) and PAS (c) staining from Piezo1^fl/fl^ and Piezo1^fl/fl^ Id2CreER^T2^ (cKO) mice treated with PBS or *A. alternata*. (Scale bars = 200 µm) (d) Representative flow cytometry plots and quantification of IL-5⁺ and IL-13⁺ ILCs (Lin⁻ CD127⁺) in lung tissue (n=7–8). (e) Quantification of total ILCs (Lin⁻ CD127⁺) in lung tissue (n=6–7). (f) Frequencies of neutrophils, eosinophils, monocytes, alveolar macrophages (AMs), and dendritic cells (DCs) in the lung, expressed as a percentage of CD45⁺ cells (n=7–13). Statistical significance was determined using one-way ANOVA. Data are pooled from at least two to three independent experiments and presented as mean ± SEM. **P < 0.01, ***P < 0.001, ****P < 0.0001; ns, not significant.

**
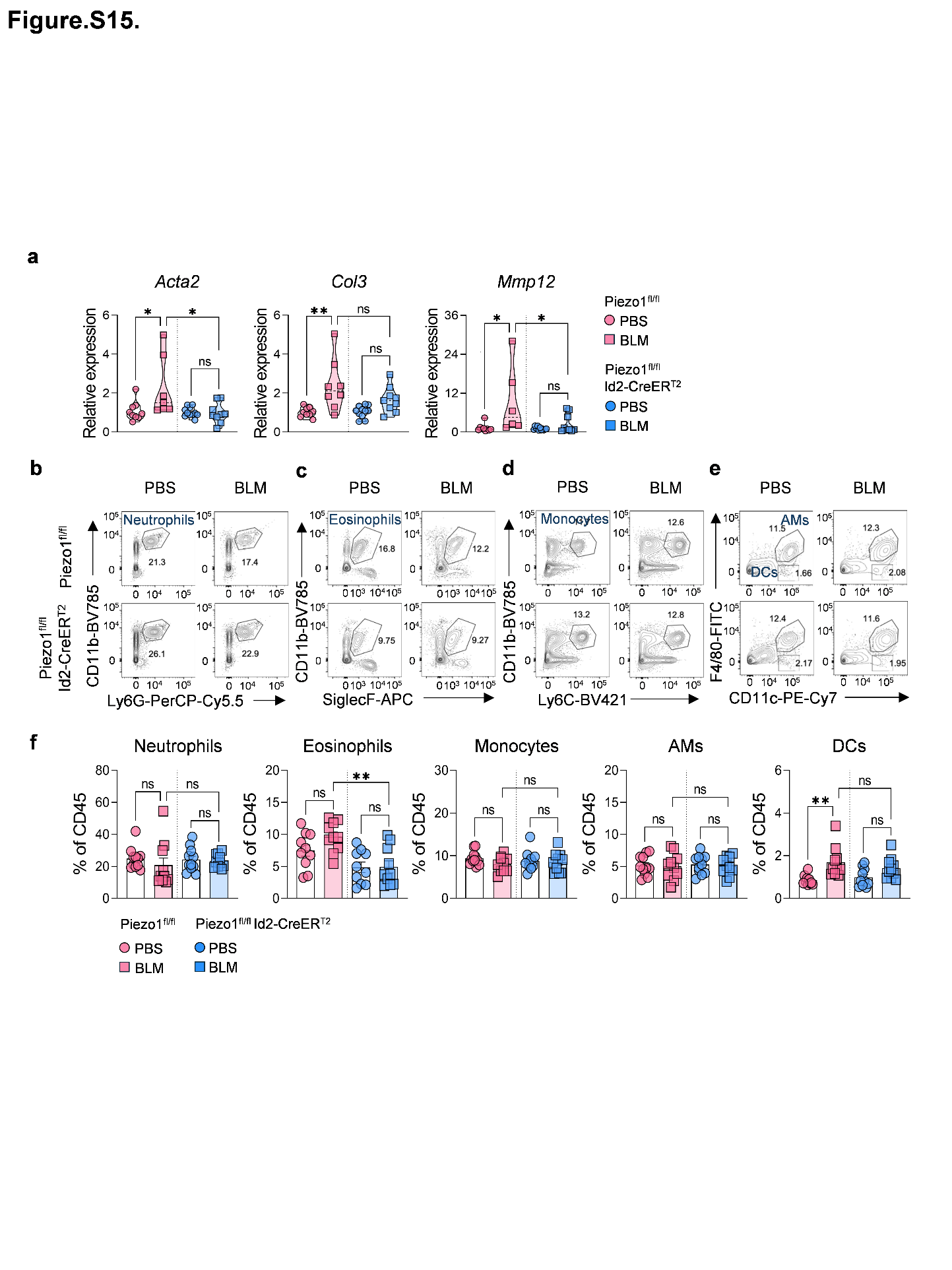
Figure. S15.**

**Supplementary Figure 15 | Myeloid cell profiling and fibrosis-related gene expression in Piezo1^fl/fl^ and Piezo1^fl/fl^ Id2CreER^T2^ (cKO) mice after bleomycin challenge.**

(a) Relative expression levels of fibrosis-associated genes (*Acta2, Col3a1, Mmp12*) in whole lung tissue from Piezo1^fl/fl^ and cKO mice treated with PBS or bleomycin (BLM), measured by qPCR (n=6–11). (b–e) Representative flow cytometry plots showing lung neutrophils (CD11b⁺ Ly6G⁺), eosinophils (CD11b⁺ SiglecF⁺), monocytes (CD11b⁺ Ly6C⁺), alveolar macrophages (F4/80⁺ CD11c⁺), and dendritic cells (DC; F4/80⁻ CD11c⁺). (f) Quantification of each myeloid population as a percentage of CD45⁺ cells in PBS- and BLM-treated mice (n=8–12). Statistical significance was determined using one-way ANOVA. Data are pooled from at least two to three independent experiments and presented as mean ± SEM. *P < 0.05, **P < 0.01; ns, not significant.


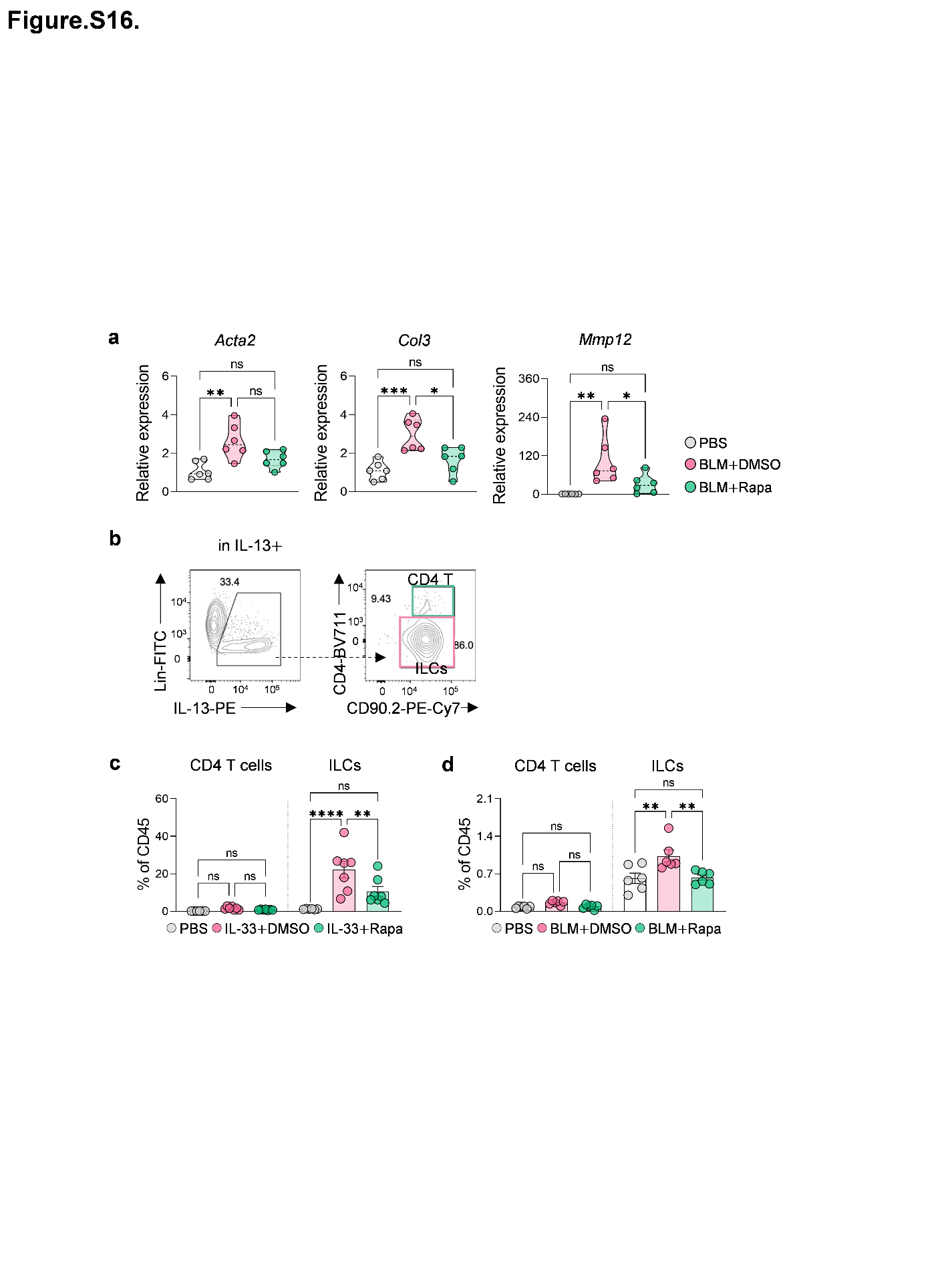
Figure. S16.

**Supplementary Fig. 16 | Rapamycin selectively reduces IL-13⁺ ILC2s and suppresses fibrosis-related gene expression.**

(a) Relative expression levels of fibrosis-associated genes (*Acta2*, *Col3a1*, and *Mmp12*) in whole lung homogenates from PBS-, bleomycin (BLM) + DMSO-, and BLM + rapamycin (Rapa) -treated mice, measured by qPCR (n=6). (b) Gating strategy for identifying IL-13⁺ CD4⁺ T cells and IL-13⁺ ILCs among lung CD45⁺ lymphocytes. (c) Frequencies of IL-13⁺ CD4⁺ T cells and IL-13⁺ ILCs in lungs of mice treated with PBS, IL-33 + DMSO, or IL-33 + Rapa (n=6–7). (d) Frequencies of IL-13⁺ CD4⁺ T cells and IL-13⁺ ILCs in BLM + DMSO vs. BLM + Rapa-treated mice (n=6). Statistical significance was determined using one-way ANOVA. Data are pooled from two to three independent experiments and presented as mean ± SEM. *P < 0.05, **P < 0.01, ***P < 0.001, ****P < 0.0001; ns, not significant.


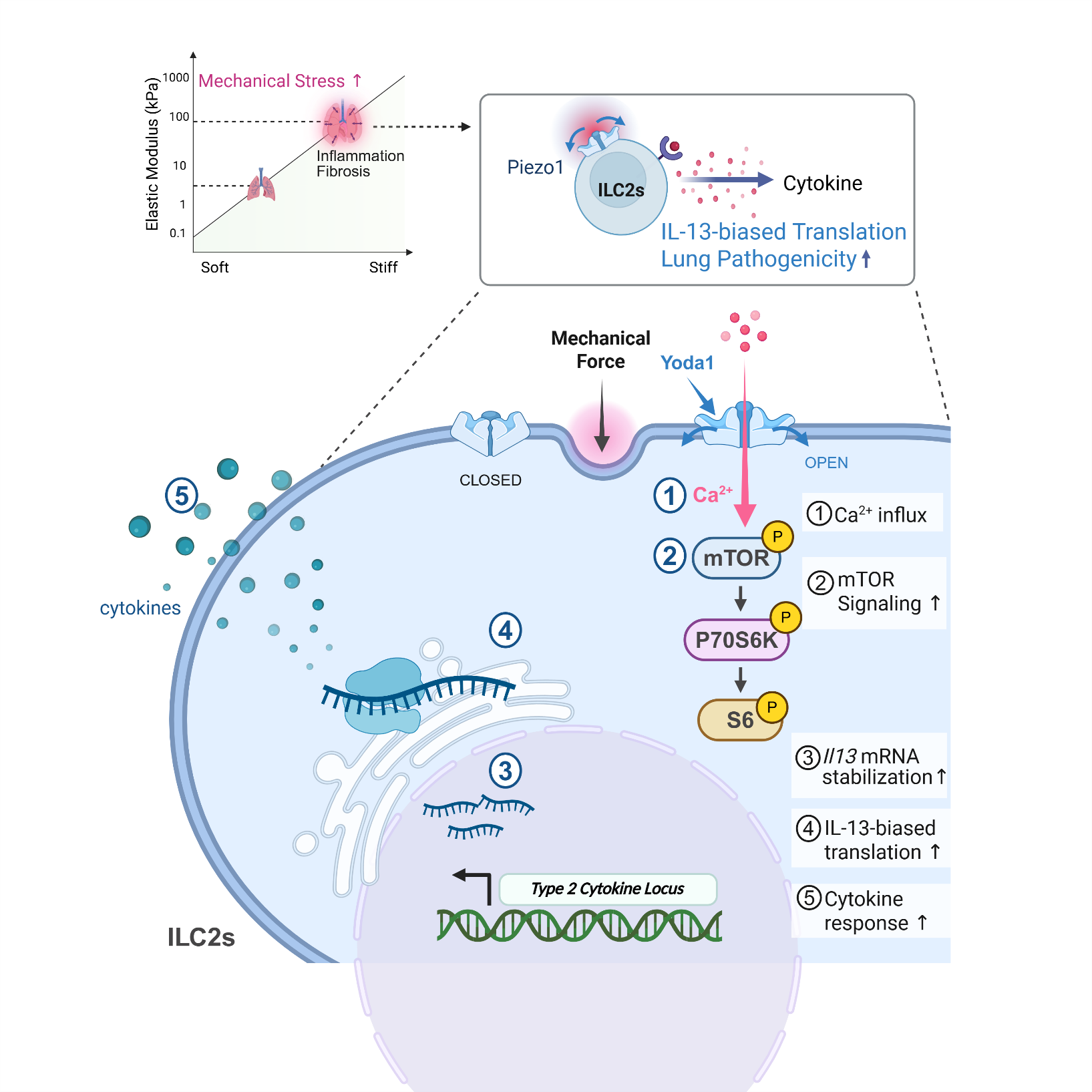
Figure. S17.

**Supplementary Fig. 17 | Schematic model of Piezo1-mediated mechanotransduction in ILC2s.**

Mechanical stress in inflamed or fibrotic lungs activates Piezo1 in ILC2s, leading to Ca²⁺ influx and mTOR pathway activation. This promotes *Il13* mRNA stabilization and IL-13–biased translation, enhancing type 2 cytokine production and contributing to lung inflammation and fibrosis.

Other Supplementary Materials for this manuscript include the following:

Captions for Movies S1 to S6

Movie S1.

Yoda1 (5$\mu$M) response in CAL-520, AM stained ILC2s without BAPTA. (Related in Figure 1d)

Movie S2.

Yoda1 (5$\mu$M) response in CAL-520, AM stained ILC2s with BAPTA. (Related in Figure 1d)

Movie S3.

Yoda1 (0$\mu$M) response in CAL-520, AM stained ILC2s. (Related in Figure S1g)

Movie S4.

Yoda1 (1$\mu$M) response in CAL-520, AM stained ILC2s. (Related in Figure S1g)

Movie S5.

Yoda1 (5$\mu$M) response in CAL-520, AM stained ILC2s. (Related in Figure S1g)

Movie S6.

Yoda1 (25$\mu$M) response in CAL-520, AM stained ILC2s. (Related in Figure S1g)
