# Supplementary figures and images for: Piezo1-mediated mechanotransduction regulates the translational activity, function and lung pathogenicity of group 2 innate lymphoid cells
Source: Signal Transduct Target Ther. 2025 Aug 21;10:269. doi: 10.1038/s41392-025-02350-4 (PMC12370950; doi:10.1038/s41392-025-02350-4)

Original and uncropped films of Western blotting (Figure. 3a, 3c, 3e, 3g, S7a, and S7b)


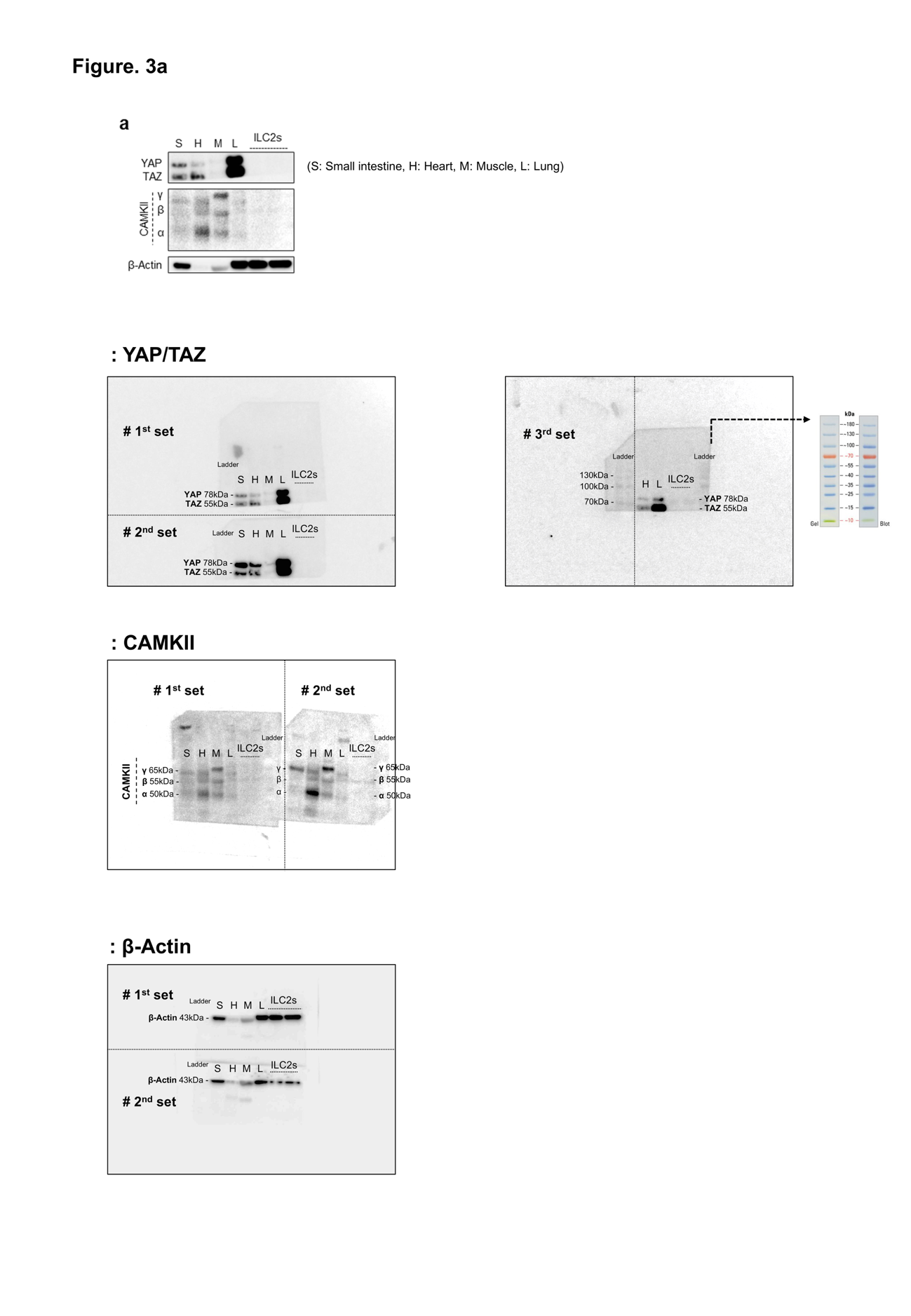


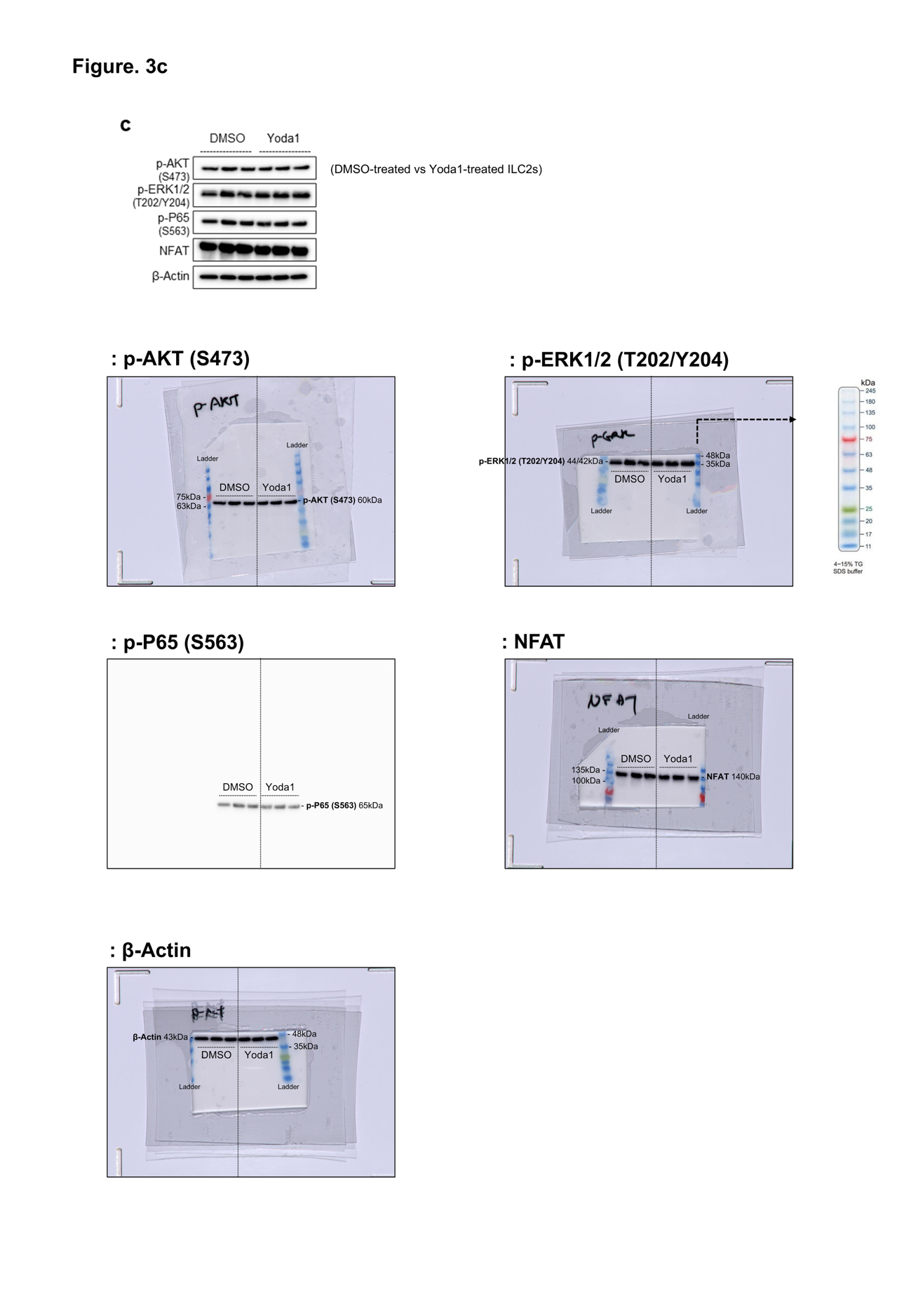

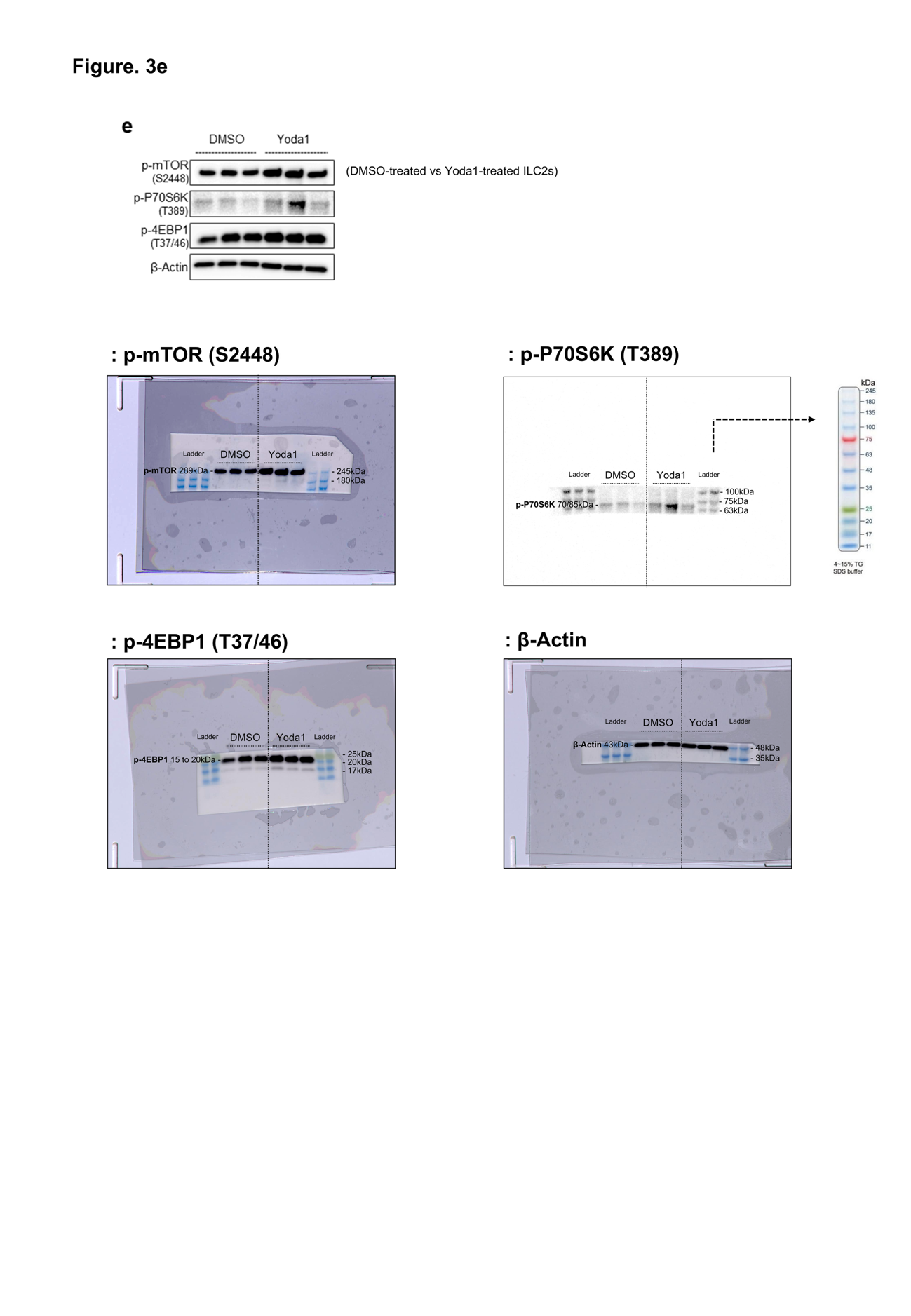


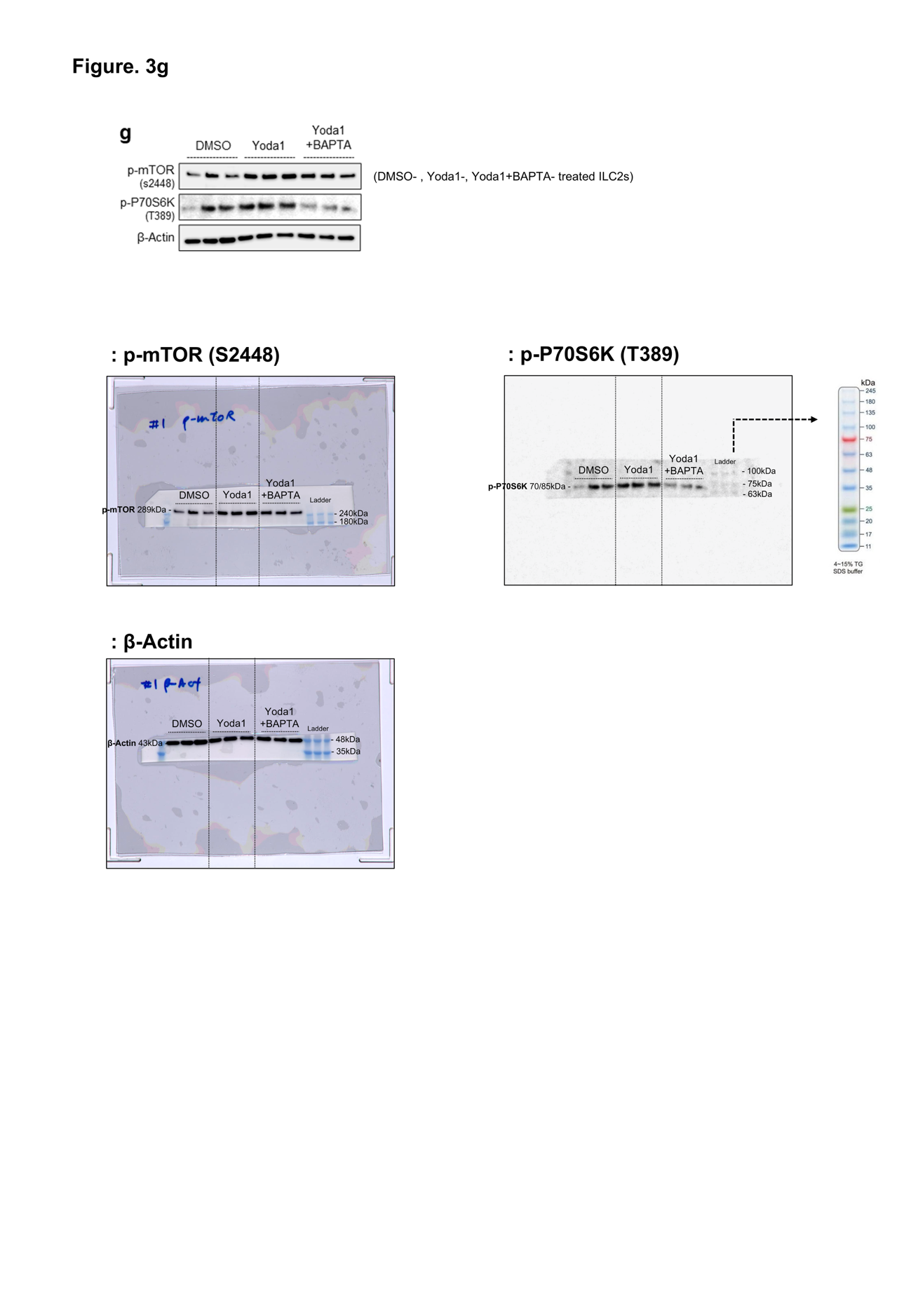

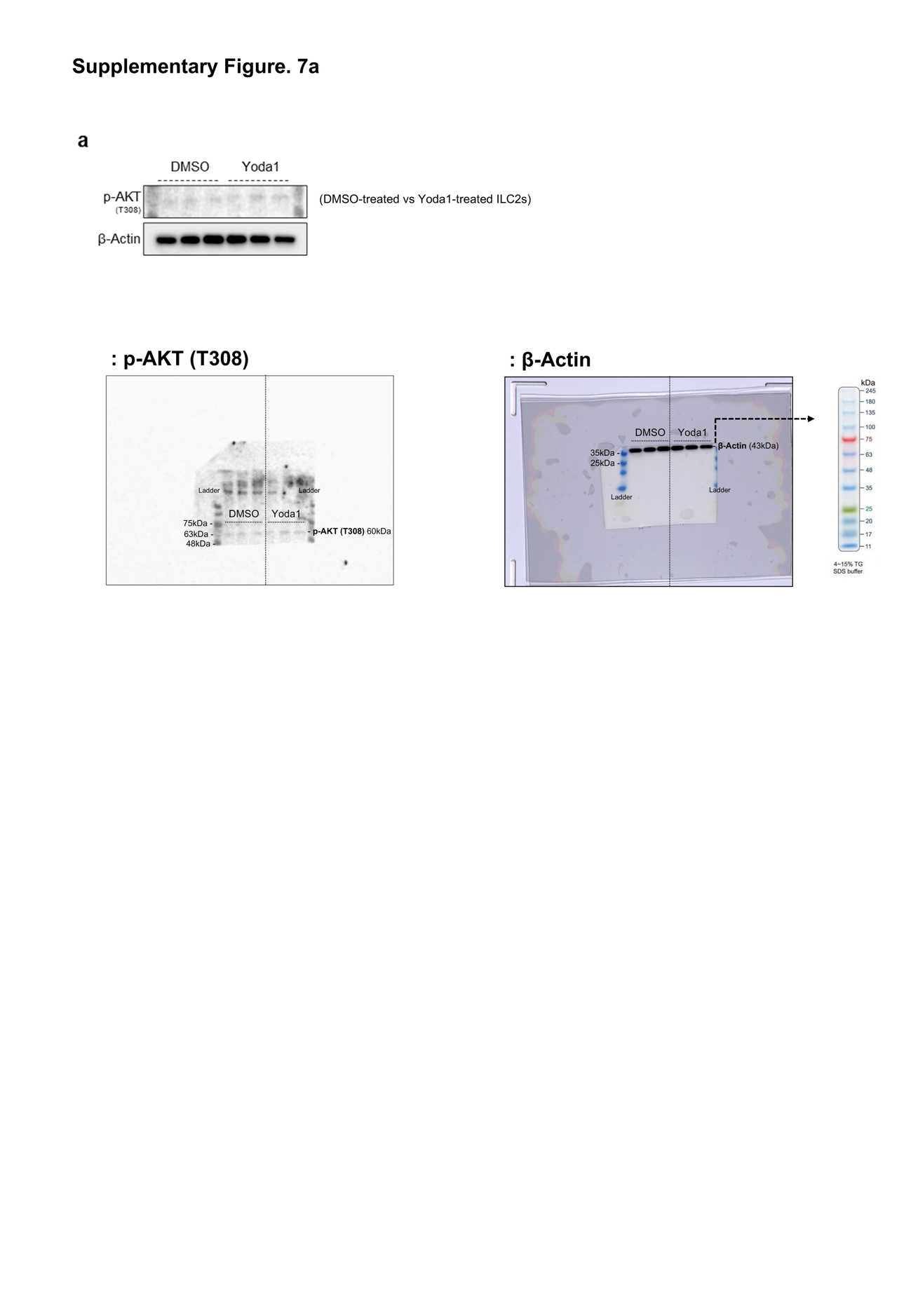


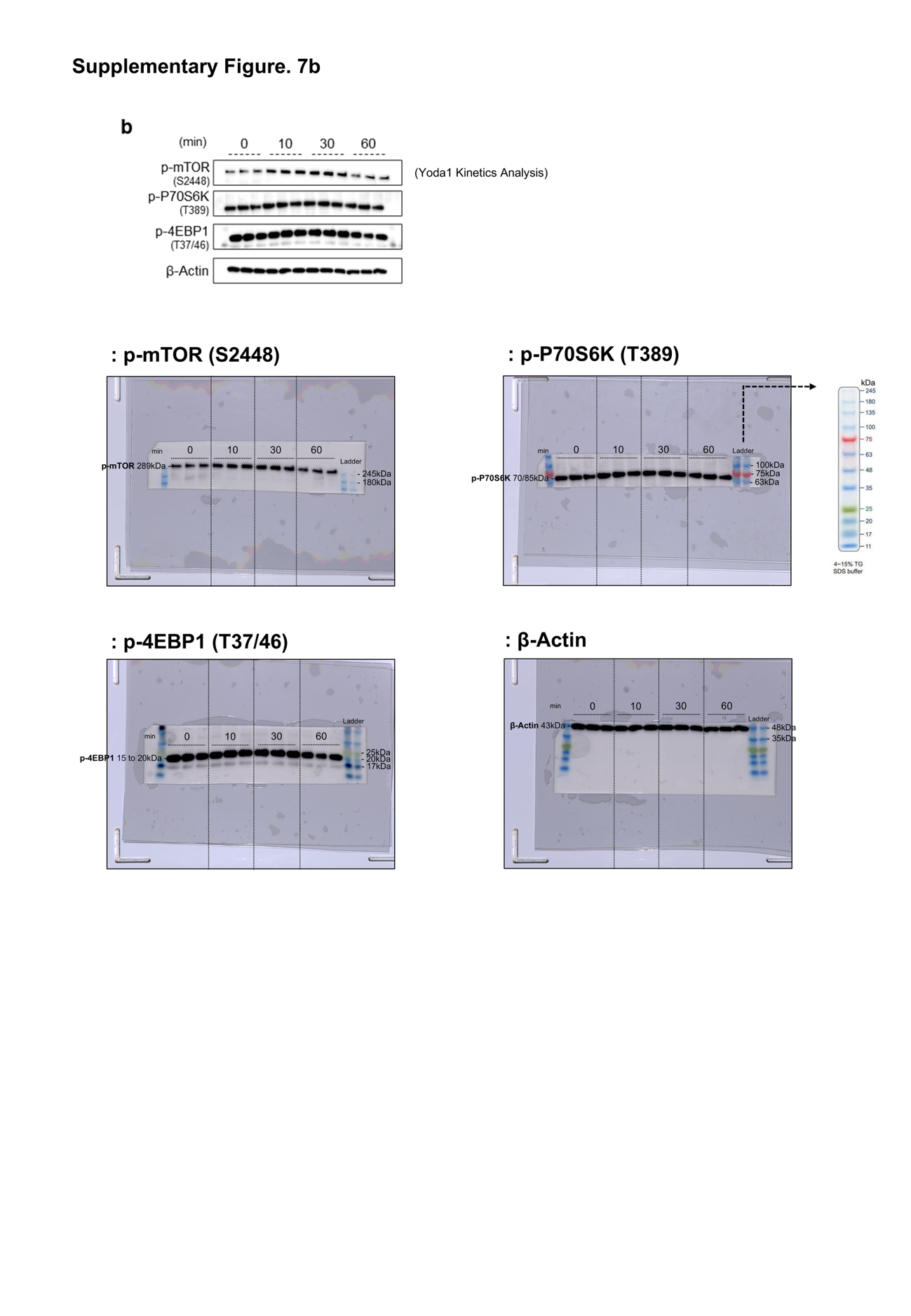

Supplement: Supplementary file 2 — Western blot original film [file 41392_2025_2350_MOESM2_ESM.docx]
